# Genomic Analysis of Influenza A and B Viruses Carrying Baloxavir Resistance-Associated Substitutions Serially Passaged in Human Epithelial Cells

**DOI:** 10.3390/v15122446

**Published:** 2023-12-16

**Authors:** Brady T. Hickerson, Bruce K. Huang, Svetlana N. Petrovskaya, Natalia A. Ilyushina

**Affiliations:** 1 Division of Biotechnology Review and Research II, Food and Drug Administration, Silver Spring, MD 20993, USA; 2 Division of Biotechnology Review and Research III, Food and Drug Administration, Silver Spring, MD 20993, USA

**Keywords:** influenza, baloxavir resistance, polymerase substitutions, viral mutation

## Abstract

Baloxavir marboxil (baloxavir) is an FDA-approved inhibitor of the influenza virus polymerase acidic (PA) protein. Here, we used next-generation sequencing to compare the genomic mutational profiles of IAV H1N1 and H3N2, and IBV wild type (WT) and mutants (MUT) viruses carrying baloxavir resistance-associated substitutions (H1N1—PA I38L, I38T, and E199D; H3N2—PA I38T; and IBV—PA I38T) during passaging in normal human bronchial epithelial (NHBE) cells. We determined the ratio of nonsynonymous to synonymous nucleotide mutations (d_N_/d_S_) and identified the location and type of amino acid (AA) substitutions that occurred at a frequency of ≥30%. We observed that IAV H1N1 WT and MUT viruses remained relatively stable during passaging. While the mutational profiles for IAV H1N1 I38L, I38T, and E199D, and IBV I38T MUTs were relatively similar after each passage compared to the respective WTs, the mutational profile of the IAV H3N2 I38T MUT was significantly different for most genes compared to H3N2 WT. Our work provides insight into how baloxavir resistance-associated substitutions may impact influenza virus evolution in natural settings. Further characterization of the potentially adaptive mutations identified in this study is needed.

## 1. Introduction

Influenza A and B viruses (IAV and IBV) can cause an acute, sometimes fatal, respiratory illness in humans. IAV and IBV are responsible for annual outbreaks of influenza resulting in upwards of 3–5 million cases of severe disease worldwide [1,2]. In addition, emergent IAV strains have caused several pandemics throughout recorded history, the largest of which occurred in 1918 and claimed millions of lives [3,4], and the most recent occurring in 2009. Two classes of antivirals are used against influenza virus infection [5]. These include the neuraminidase (NA) inhibitors (oseltamivir carboxylate, zanamivir, peramivir, and laninamivir) and the polymerase acidic (PA) inhibitor baloxavir marboxil (active form: baloxavir acid; baloxavir) [5].

The effectiveness of FDA-approved influenza virus antivirals can be compromised by the development of resistance [6]. Influenza viruses exist as quasispecies due to spontaneous nucleotide mutations introduced in the virus genome during replication due to low polymerase fidelity [7,8,9]. While most spontaneous nucleotide mutations result in synonymous or nonsynonymous amino acid (AA) substitutions that have deleterious consequences for viral replication capacity, some of the mutations may code for AA substitutions that are beneficial, including by conferring resistance to antiviral inhibition [10,11,12,13,14,15,16,17]. Periodically, influenza viruses have emerged in widespread circulation and have displayed resistance to antiviral drugs. For example, influenza strains resistant to M2-ion channel inhibitors are currently widespread and have resulted in the discontinued use of these antivirals [18]. Furthermore, the emergence and widespread circulation of oseltamivir-resistant H1N1 in 2007–2009, although subsequently replaced by a susceptible IAV H1N1 strain, has generated persistent concern for the continued effectiveness of these countermeasures if resistant mutants should become widespread again [19,20]. Resistance to baloxavir is most often mediated by AA substitutions at the conserved isoleucine position 38 of the influenza PA protein (i.e., I38F/L/M/S/T), but AA substitutions at E23G/K/R, A36V, A37E, E119D/G, E198K, and E199D in IAV H1N1; E23K, A36V, A37T, and E119D/G in IAV H3N2; and E23K, A36V, L28P, A36V, E119G, and G199R in IBV have also been reported [12,15,21,22,23,24,25,26,27,28]. Continuous monitoring and assessment of drug resistance-associated substitutions are warranted for the effective use of baloxavir to treat influenza virus infections.

Next-generation sequencing (NGS) is a powerful sequencing technology for influenza virus surveillance [29]. Previously, NGS has been employed to track outbreaks of NA inhibitor-resistant influenza variants [30]. Moreover, NGS was used to identify additional substitutions within viral surface proteins that compensated for the replication deficits induced by the NA resistance-associated substitutions [31]. Analysis of NGS data from recent clinical trials identified several AA substitutions within influenza viruses collected from individuals who underwent baloxavir treatment [12]. In addition, our recent study using NGS identified novel PA substitutions (i.e., D394N and E329G) that arose after passaging in A/California/04/09 (H1N1) and B/Victoria/504/2000 viruses carrying baloxavir resistance-associated substitutions PA I38L and I38T, respectively [13].

To better understand the genomic evolution of influenza viruses, we serially passaged A/California/04/09 (H1N1)-, A/Hong Kong/218849/2006 (H3N2)-, and B/Victoria/504/2000 (IBV)-like drug-sensitive wild-type (WT) and drug-resistant mutant (MUT) viruses (i.e., IAV H1N1 PA I38L, I38T, and E199D; IAV H3N2 PA I38T; and IBV PA I38T) in normal human bronchial epithelial (NHBE) cells and then analyzed their genomes for the emergence of mutations. We compared the genomic mutation profiles of the MUT viruses to their respective WT virus counterpart after each passage, determined the total number and type of mutations, and the ratio of nonsynonymous to synonymous mutations (d_N_/d_S_) for each viral gene after each passage. We mapped the AA substitutions that occurred at frequencies of ≥30% on representative 3D structures of the respective viral protein.

## 2. Materials and Methods

### 2.1. Cells

Madin–Darby canine kidney (MDCK) and human embryonic kidney (293T) cells were obtained from the American Tissue Culture Collection (Manassas, VA, USA) and were maintained as previously described [32]. Primary NHBE cells were obtained from Lonza (Walkersville, MD, USA) and were grown on 6.5 mm Transwell membrane supports (Corning Inc., Corning, NY, USA) at the air–liquid interface in serum-free and hormone- and growth factor-supplemented medium, as described previously [33]. Once confluent, the NHBE cells were allowed to fully differentiate for at least 3 weeks and were used in all the experiments after mucin production was observed on the apical side.

### 2.2. Viruses

Recombinant influenza A/California/04/09 (H1N1), A/Hong Kong/218849/2006 (H3N2), and B/Victoria/504/2000 (IBV) viruses were generated by DNA transfection of 293T cells [34]. Point mutations to induce amino acid substitutions in the IAV H1N1 PA (I38L, I38T, or E199D), H3N2 PA (I38T), or IBV PA (I38T) genes of the respective baloxavir-sensitive viruses were introduced using the Quick-change site-directed mutagenesis kit (Stratagene, La Jolla, CA, USA). Stocks of drug-sensitive (i.e., IAV H1N1 and H3N2, and IBV WT) and drug-resistant (i.e., IAV H1N1 I38L, H1N1 I38T, H1N1 E199D, and H3N2 I38T, and IBV I38T) MUT viruses were prepared by incubation of the viruses in 10-day-old embryonated chicken eggs for 48 h at 37 °C for IAV H1N1 and H3N2 or 72 h at 33 °C for IBV, respectively. All genes of rescued viruses were analyzed by Sanger sequencing and NGS to verify the presence of the desired PA mutations and the absence of additional mutations. The virus stocks were quantified by titration in MDCK cells. Briefly, confluent cultures of MDCK cells were incubated for 1 h with 10-fold serial dilutions of virus mixtures or samples at 37 °C for IAV H1N1 and H3N2 or 33 °C for IBV. The cells were then washed and overlaid with minimal essential medium containing 0.3% bovine serum albumin (BSA), 0.25% agarose, and 1 μg/mL l-(tosylamido-2-phenyl)ethylchloromethylketone (TPCK)-treated trypsin. After 2 days of incubation at 37 °C for IAV H1N1 and H3N2 or 3 days of incubation at 33 °C for IBV, the cells were stained with 0.1% crystal violet in 10% formaldehyde solution and the number of plaque-forming units (PFU)/mL was determined. All experimental work was performed in a biosafety level-2 laboratory approved for the use of the IAV and IBV strains by the U.S. Department of Agriculture and the U.S. Centers for Disease Control and Prevention.

### 2.3. Viral Sequence Analysis

Sanger sequencing and NGS were performed on all viral genes of the virus stocks to detect the presence of the desired mutations and the absence of other mutations. NGS was also used to detect the presence of mutations after serial passaging of the viruses in NHBE cells. Viral RNA was extracted from virus stocks using the Qiagen RNeasy Mini kit (Germantown, MD, USA). For Sanger sequencing, samples were then reverse transcribed and analyzed by polymerase chain reaction (PCR) using universal primers specific for the viral genes, as described previously [35]. The Sanger sequencing was performed by the Research Central Facility for Biotechnology Resources at the U.S. Food and Drug Administration (Silver Spring, MD, USA). DNA sequences were completed, edited, and analyzed using the DNASTAR Laser gene sequence analysis software package version 17 (Madison, WI, USA). For NGS, RNA libraries were prepared from the extracted viral RNA samples using the NEBNext Ultra II RNA Library Prep Kit for Illumina (New England BioLabs, Ipswich, MA, USA). Fragmentation and priming of the samples were performed in one reaction with the fragmentation buffer and random primer mix provided in the kit. The double-stranded cDNA synthesis and the library construction were carried out according to the manufacturer’s protocol. The libraries were analyzed for size distribution and concentration with TapeStation 4200 (Agilent Technologies, Santa Clara, CA, USA) and Qubit 4 (Invitrogen, Waltham, MA, USA), respectively. NGS was performed using NextSeq 500 (Illumina, San Diego, CA, USA) to produce 2 × 75 nt PE reads. The raw sequencing reads were then analyzed by using the FDA in-house developed High-performance Integrated Virtual Environment (HIVE; [36]). The resulting runs produced an average read depth of ≥1800 for each nucleotide position with each influenza virus gene. Only mutations that were present at 5% or greater at nucleotide positions with a read depth of ≥1500 were considered to rule out any false mutations that may be due to sequencing artifacts or background noise. The resulting data were aligned to the viral gene sequence of the respective virus and then used to determine the overall frequency of mutations in the viral gene, the similarity of the WT and MUT virus mutation profiles, if the observed mutations were nonsynonymous or synonymous, and the location of the resulting AA substitutions in each virus protein. The rates of nonsynonymous and synonymous mutations within each viral gene for each passage were used to calculate the d_N_/d_S_ ratios.

### 2.4. Serial Passaging

IAV H1N1 and H3N2, and IBV WT and MUT viruses were serially passaged 3 times in NHBE cells. Confluent and differentiated NHBE cells were initially infected at a multiplicity of infection of 0.1 PFU/cell (~6000 PFU) and incubated at 37 °C for IAV H1N1 and H3N2 or 33 °C for IBV for 1 h. The virus mixture was then removed, the cells were washed and then incubated at 37 °C for IAV H1N1 and H3N2 or 33 °C for IBV. After 48 h, virus-containing supernatant samples were collected from the cells and either passaged onto fresh NHBE cells (~10,000 PFU) or stored at −80 °C until use. Viral titers were determined after each passage to ensure that similar PFUs were used, and viral titers were ~10^6^–10^7^ PFU/mL, as was reported previously by our group [26]. RNA was extracted from the cell culture supernatant samples and then analyzed by NGS.

### 2.5. Mapping of AA Substitutions 

Structural bioinformatics was used to map the location of AA substitutions that arose during serial passaging of the IAV and IBV WTs and MUTs. The X-ray and cryo-EM crystal structures of the H1N1, H3N2, and IBV proteins were downloaded from the protein databank (www.rcsb.org) and analyzed using Pymol version 2.5.5 (Schrödinger, Inc., New York, NY, USA). We used the following 3D protein structures to map the AA substitutions that occurred at frequencies ≥30%: H1N1 PB2—7NHX; H3N2 PB2, PB1, and PA—6QX3, HA—7K37, NA—6BR5, and NS—3EE8; IBV PB2 and PA—5FMZ. These structures were chosen because they are the structures of the respective virus isolate or because they have a high degree of AA sequence similarity with the viruses used in the study. The baloxavir-resistant AA substitutions were also mapped onto the PA proteins of the respective MUTs and colored in red to distinguish these sites from the rest of the structure. Residue substitutions that arose during serial passaging were mapped in blue. Baloxavir acid binding to the influenza virus PA endonuclease domain was colored yellow. Protein domains with specific functions were colored light blue. The known binding locations of the viral proteins are shown in green.

### 2.6. Statistical Analysis

The mutational profiles of the serially passaged viruses shown in Figures 1–8 were compared using the unpaired *t*-test (Prism 9.0, GraphPad Software, La Jolla, CA, USA). Probability values of ≤0.05 indicated statistically significant differences.

## 3. Results

### 3.1. Serial Passaging of the WT and MUT Viruses and Alignment of NGS Reads to the Influenza Virus Genome

**Figure 1:**
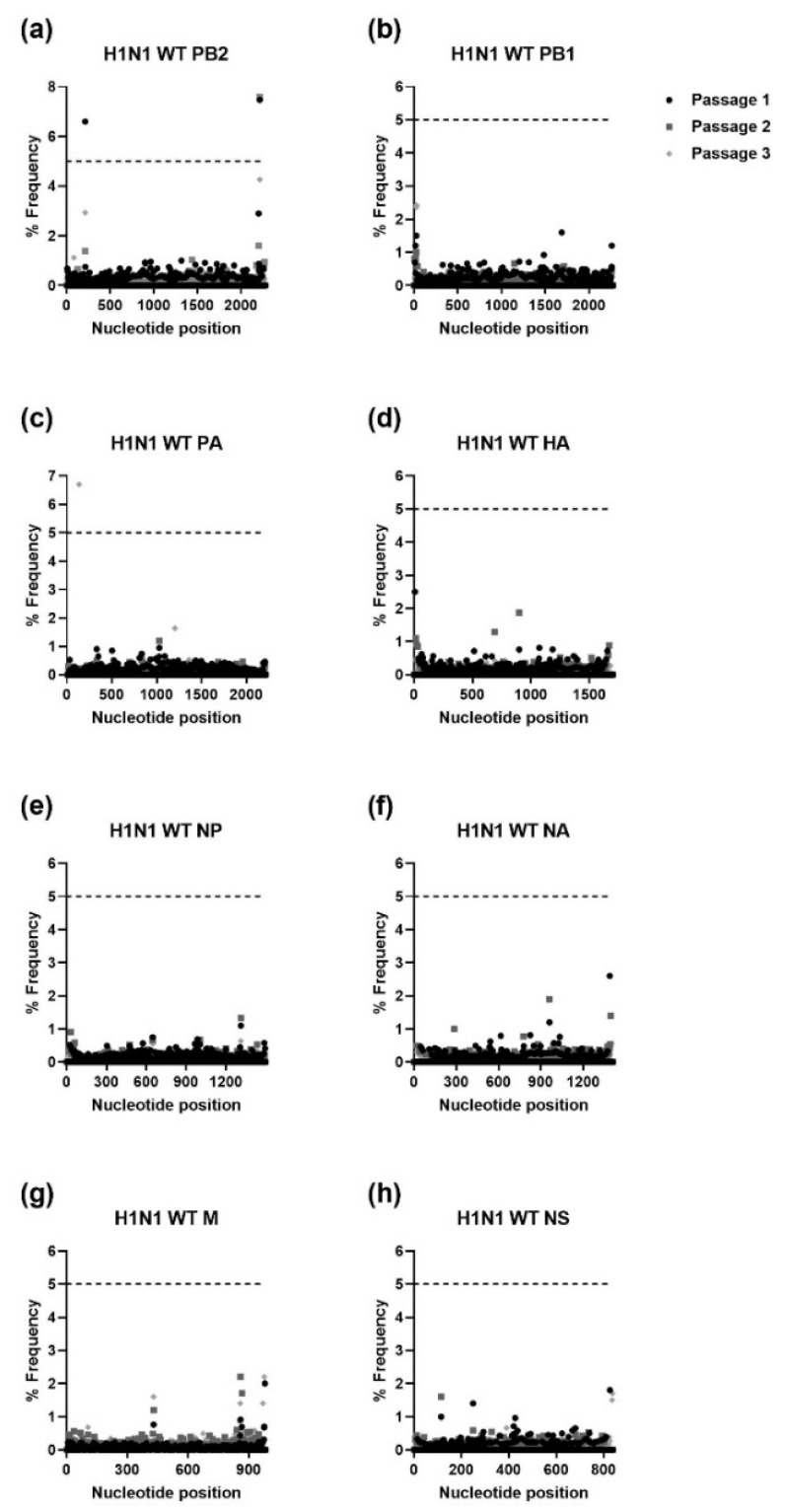
Percent frequency of mutations at each nucleotide loci for each passage within the IAV H1N1 WT genes. ((**a**) PB2, (**b**) PB1, (**c**) PA, (**d**) HA, (**e**) NP, (**f**) NA, (**g**) M, (**h**) NS). To generate mutations, H1N1 WT virus was serially passaged three times in NHBE cells. NGS was then performed on RNA extracted from supernatant aliquots from each passage and the data were analyzed using the FDA in-house NGS data analysis platform, HIVE. The dashed line indicates the 5% cutoff mutation frequency. PB2, Polymerase basic 2; PB1, Polymerase basic 1; PA, Polymerase acidic; HA, Hemagglutinin; NP, Nucleoprotein; NA, Neuraminidase; M, Matrix; NS, Nonstructural.

**Figure 2:**
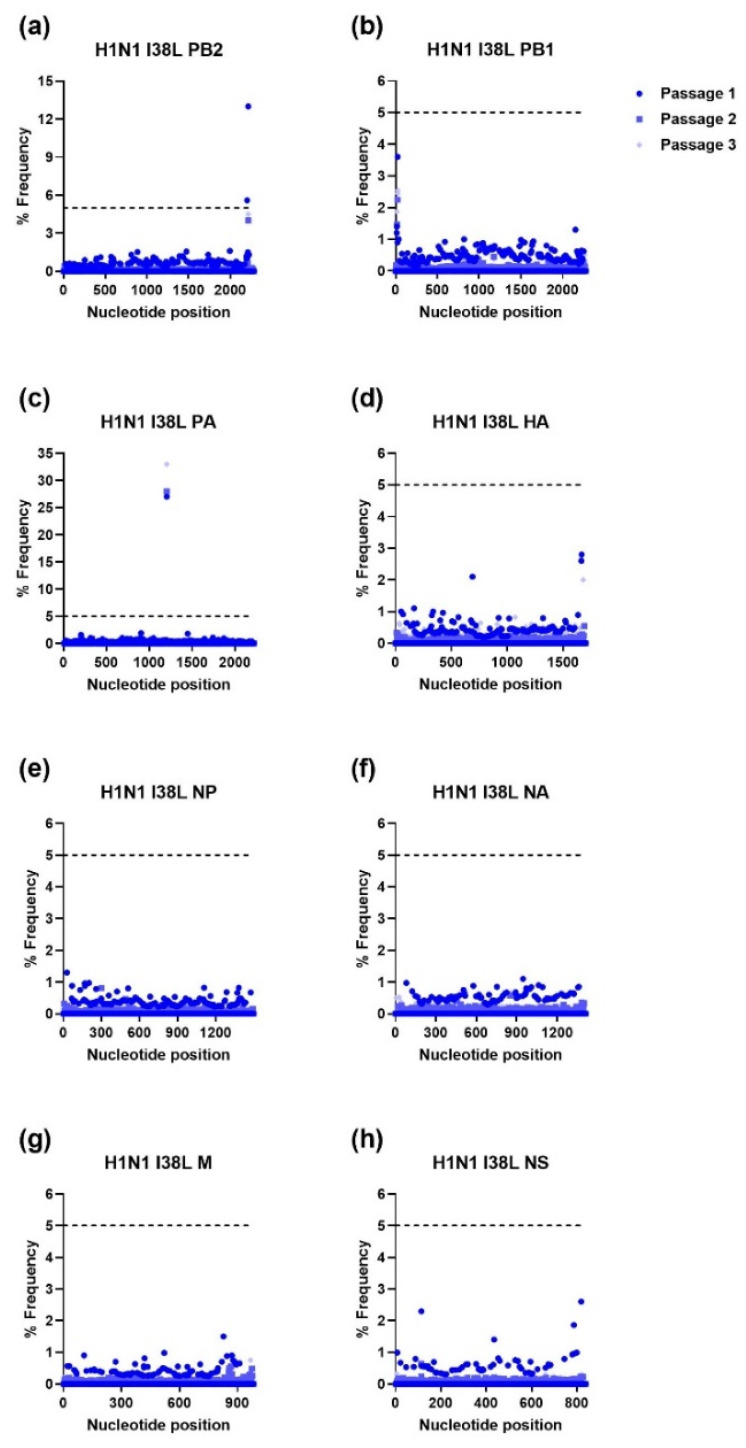
Percent frequency of mutations at each nucleotide loci for each passage within the IAV H1N1 I38L genes ((**a**) PB2, (**b**) PB1, (**c**) PA, (**d**) HA, (**e**) NP, (**f**) NA, (**g**) M, (**h**) NS). To generate mutations, H1N1 I38L MUT virus was serially passaged three times in NHBE cells. NGS was then performed on RNA extracted from supernatant aliquots from each passage and the data were analyzed using the FDA in-house NGS data analysis platform, HIVE. The dashed line indicates the 5% cutoff mutation frequency. PB2, Polymerase basic 2; PB1, Polymerase basic 1; PA, Polymerase acidic; HA, Hemagglutinin; NP, Nucleoprotein; NA, Neuraminidase; M, Matrix; NS, Nonstructural.

**Figure 3:**
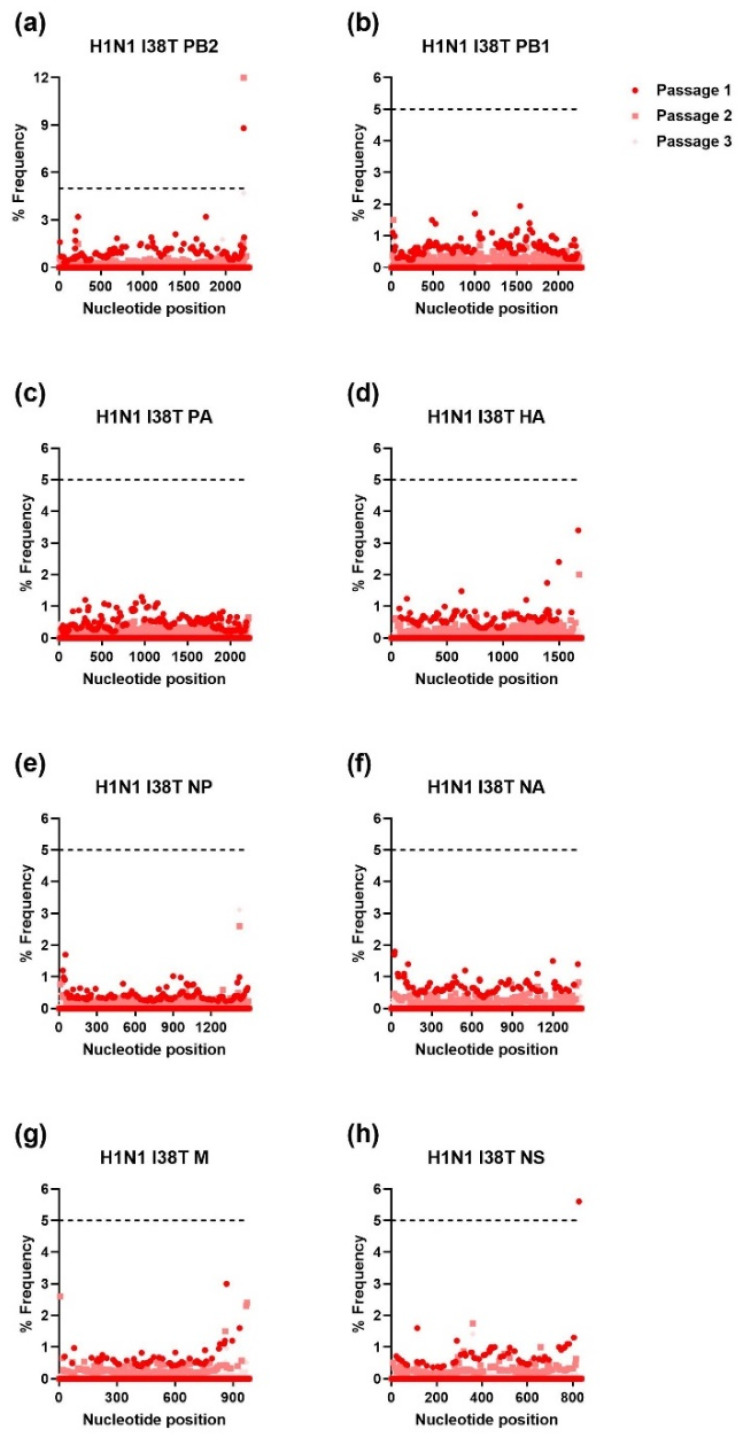
Percent frequency of mutations at each nucleotide loci for each passage within the IAV H1N1 I38T genes ((**a**) PB2, (**b**) PB1, (**c**) PA, (**d**) HA, (**e**) NP, (**f**) NA, (**g**) M, (**h**) NS). To generate mutations, H1N1 I38T MUT virus was serially passaged three times in NHBE cells. NGS was then performed on RNA extracted from supernatant aliquots from each passage and the data was analyzed using the FDA in-house NGS data analysis platform, HIVE. The dashed line indicates the 5% cutoff mutation frequency. PB2, Polymerase basic 2; PB1, Polymerase basic 1; PA, Polymerase acidic; HA, Hemagglutinin; NP, Nucleoprotein; NA, Neuraminidase; M, Matrix; NS, Nonstructural.

**Figure 4:**
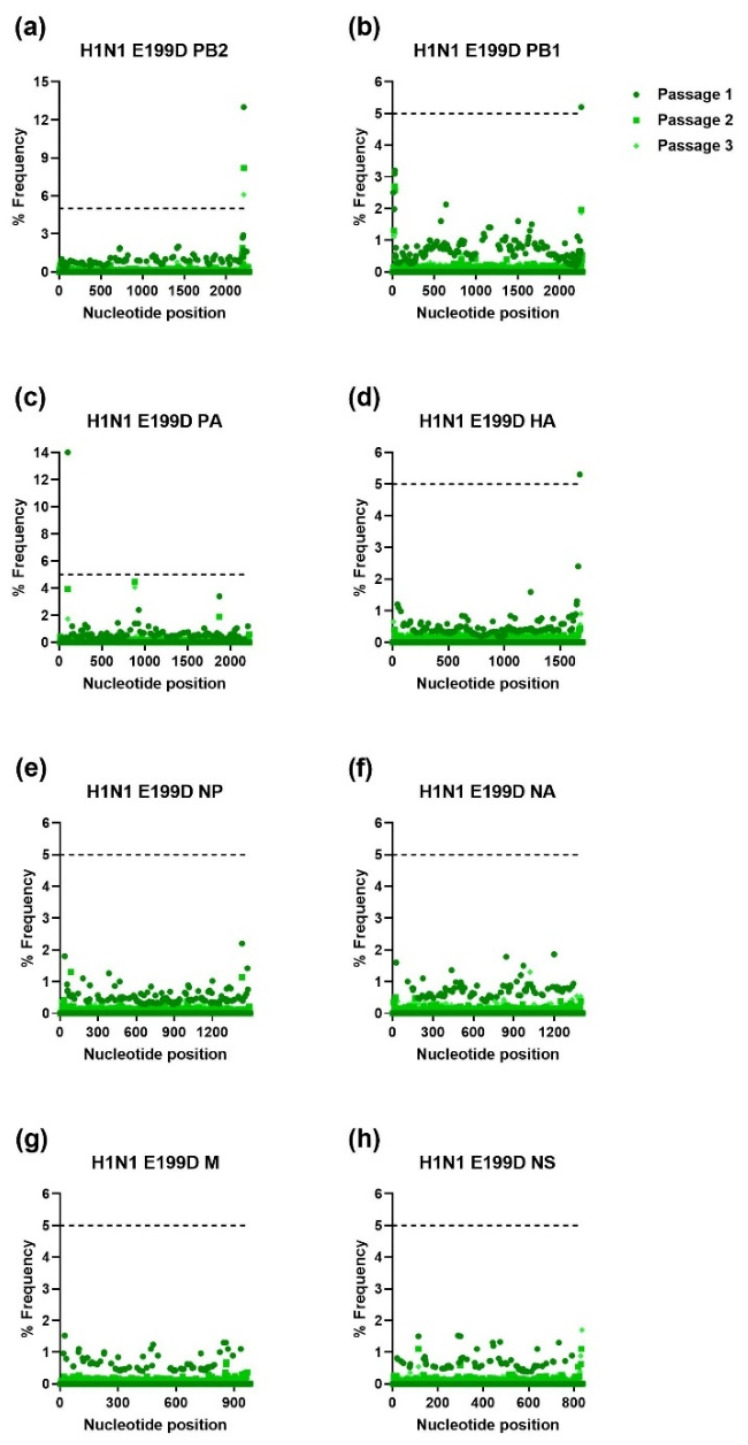
Percent frequency of mutations at each nucleotide loci for each passage within the IAV H1N1 E199D genes ((**a**) PB2, (**b**) PB1, (**c**) PA, (**d**) HA, (**e**) NP, (**f**) NA, (**g**) M, (**h**) NS). To generate mutations, H1N1 E199D MUT virus was serially passaged three times in NHBE cells. NGS was then performed on RNA extracted from supernatant aliquots from each passage and the data was analyzed using the FDA in-house NGS data analysis platform, HIVE. The dashed line indicates the 5% cutoff mutation frequency. PB2, Polymerase basic 2; PB1, Polymerase basic 1; PA, Polymerase acidic; HA, Hemagglutinin; NP, Nucleoprotein; NA, Neuraminidase; M, Matrix; NS, Nonstructural.

The IAV H1N1 and H3N2 and IBV WTs and MUTs were passaged three times in NHBE cells. RNA was extracted from supernatant samples collected after each passage and was used for NGS. The NGS sequencing was aligned to the viral stocks’ sequence for each gene of the respective WT virus and analyzed using HIVE [36]. To avoid sequencing error, only mutations that were present at a frequency of ≥5% were further analyzed. As shown in Figure 1, Figure 2, Figure 3 and Figure 4, serial passage of the IAV H1N1 WT and MUTs resulted in very few mutations that were ≥5%. Overall, the IAV H1N1 WT and MUTs remained relatively stable throughout the passages and developed only a handful of mutations during passaging. All of the H1N1 viruses developed at least one nucleotide mutation in the polymerase basic 2 (PB2) gene. The IAV H1N1 WT and I38L MUT also developed additional mutations in the PA gene. For the IAV H1N1 I38T MUT, a single mutation was observed in the nonstructural (NS) gene that was no longer detected after the first passage. Similarly, the IAV H1N1 E199D MUT developed additional mutations in the polymerase basic 1 (PB1) and hemagglutinin (HA) genes, after the first passage, which was no longer detectable after the second passage.

**Figure 5:**
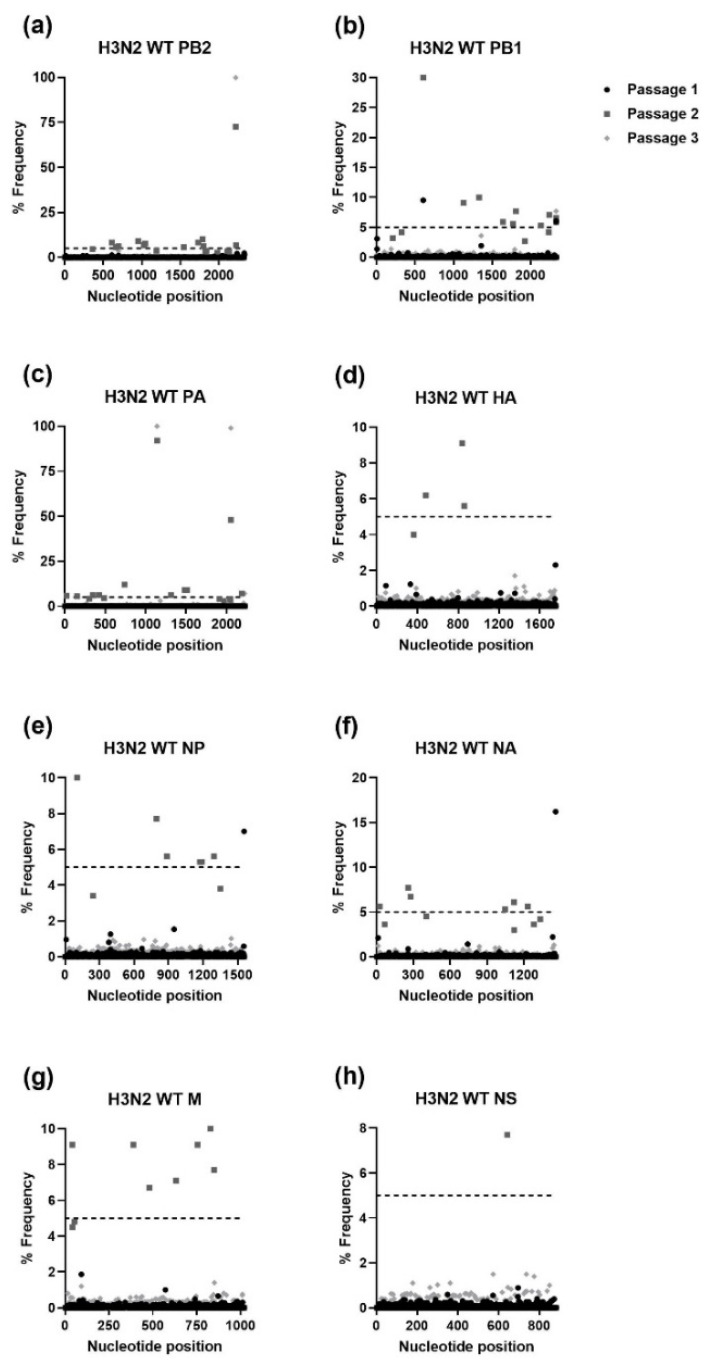
Percent frequency of mutations at each nucleotide loci for each passage within the IAV H3N2 WT genes ((**a**) PB2, (**b**) PB1, (**c**) PA, (**d**) HA, (**e**) NP, (**f**) NA, (**g**) M, (**h**) NS). To generate mutations, H3N2 WT virus was serially passaged three times in NHBE cells. NGS was then performed on RNA extracted from supernatant aliquots from each passage and the data was analyzed using the FDA in-house NGS data analysis platform, HIVE. The dashed line indicates the 5% cutoff mutation frequency. PB2, Polymerase basic 2; PB1, Polymerase basic 1; PA, Polymerase acidic; HA, Hemagglutinin; NP, Nucleoprotein; NA, Neuraminidase; M, Matrix; NS, Nonstructural.

**Figure 6:**
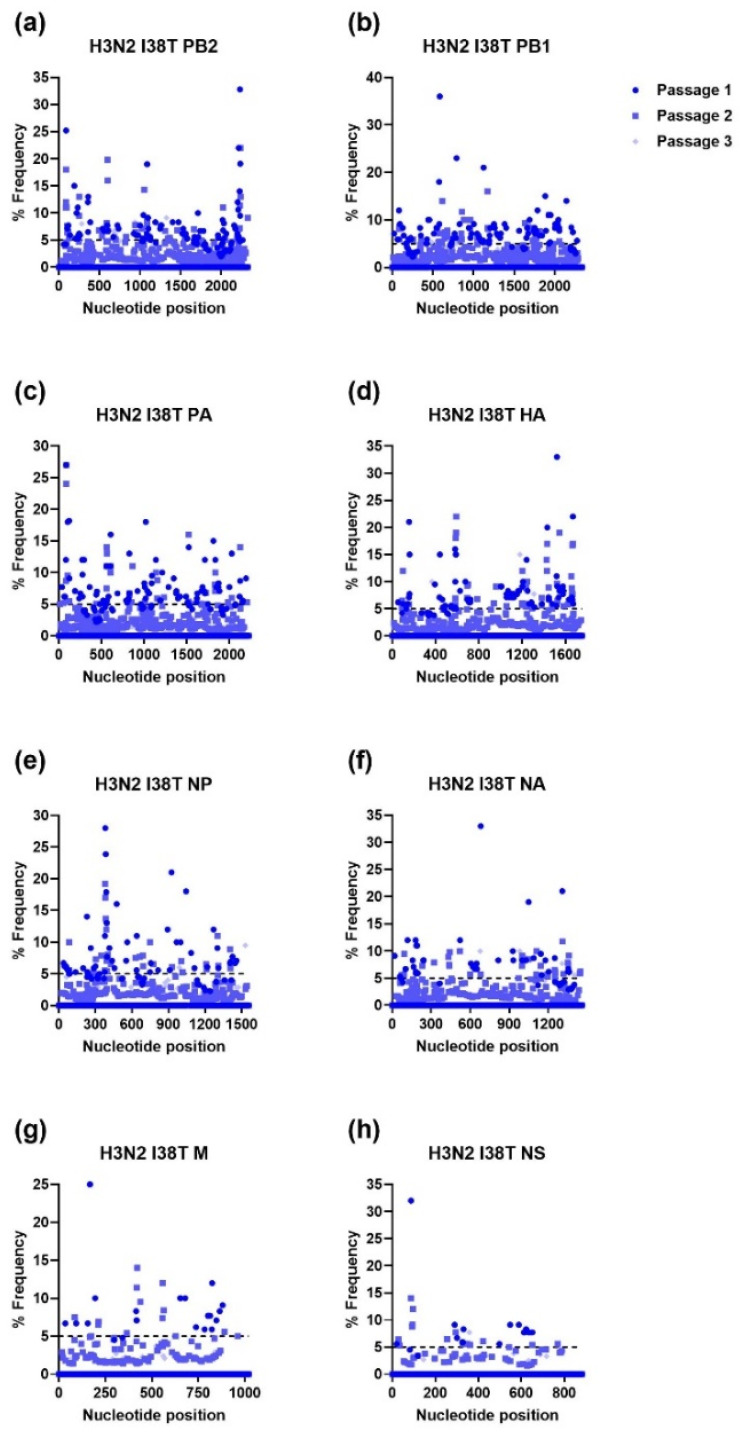
Percent frequency of mutations at each nucleotide loci for each passage within the IAV H3N2 I38T genes ((**a**) PB2, (**b**) PB1, (**c**) PA, (**d**) HA, (**e**) NP, (**f**) NA, (**g**) M, (**h**) NS). To generate mutations, H3N2 I38T MUT virus was serially passaged three times in NHBE cells. NGS was then performed on RNA extracted from supernatant aliquots from each passage and the data was analyzed using the FDA in-house NGS data analysis platform, HIVE. The dashed line indicates the 5% cutoff mutation frequency. PB2, Polymerase basic 2; PB1, Polymerase basic 1; PA, Polymerase acidic; HA, Hemagglutinin; NP, Nucleoprotein; NA, Neuraminidase; M, Matrix; NS, Nonstructural.

Unlike IAV H1N1, serial passage of the IAV H3N2 WT and I38T MUT resulted in numerous mutations in all viral genes except for the WT NS gene (Figure 5 and Figure 6). Notably, the IAV H3N2 I38T MUT had more mutations in every gene after every passage compared to the IAV H3N2 WT. The passage of the IAV H3N2 WT resulted in several mutations in PB2 and PB1 reaching frequencies approaching 100% by the third passage. For the IAV H3N2 I38T MUT, mutations were present in every gene at frequencies of >25% after the first passage, which subsequently decreased in prevalence after the second and third passages.

**Figure 7:**
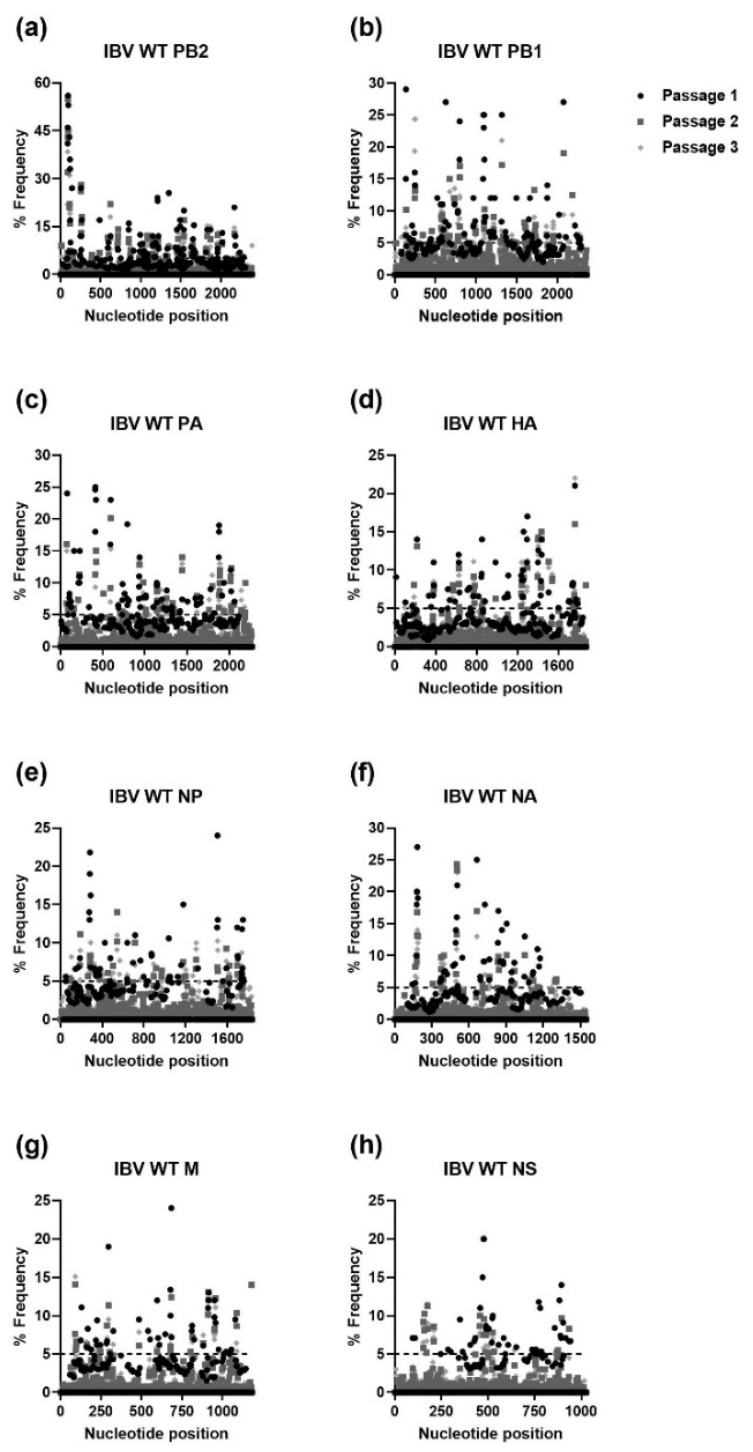
Percent frequency of mutations at each nucleotide loci for each passage within the IBV WT genes ((**a**) PB2, (**b**) PB1, (**c**) PA, (**d**) HA, (**e**) NP, (**f**) NA, (**g**) M, (**h**) NS). To generate mutations, IBV WT virus was serially passaged three times in NHBE cells. NGS was then performed on RNA extracted from supernatant aliquots from each passage and the data was analyzed using the FDA in-house NGS data analysis platform, HIVE. The dashed line indicates the 5% cutoff mutation frequency. IBV, Influenza B virus; PB2, Polymerase basic 2; PB1, Polymerase basic 1; PA, Polymerase acidic; HA, Hemagglutinin; NP, Nucleoprotein; NA, Neuraminidase; M, Matrix; NS, Nonstructural.

**Figure 8:**
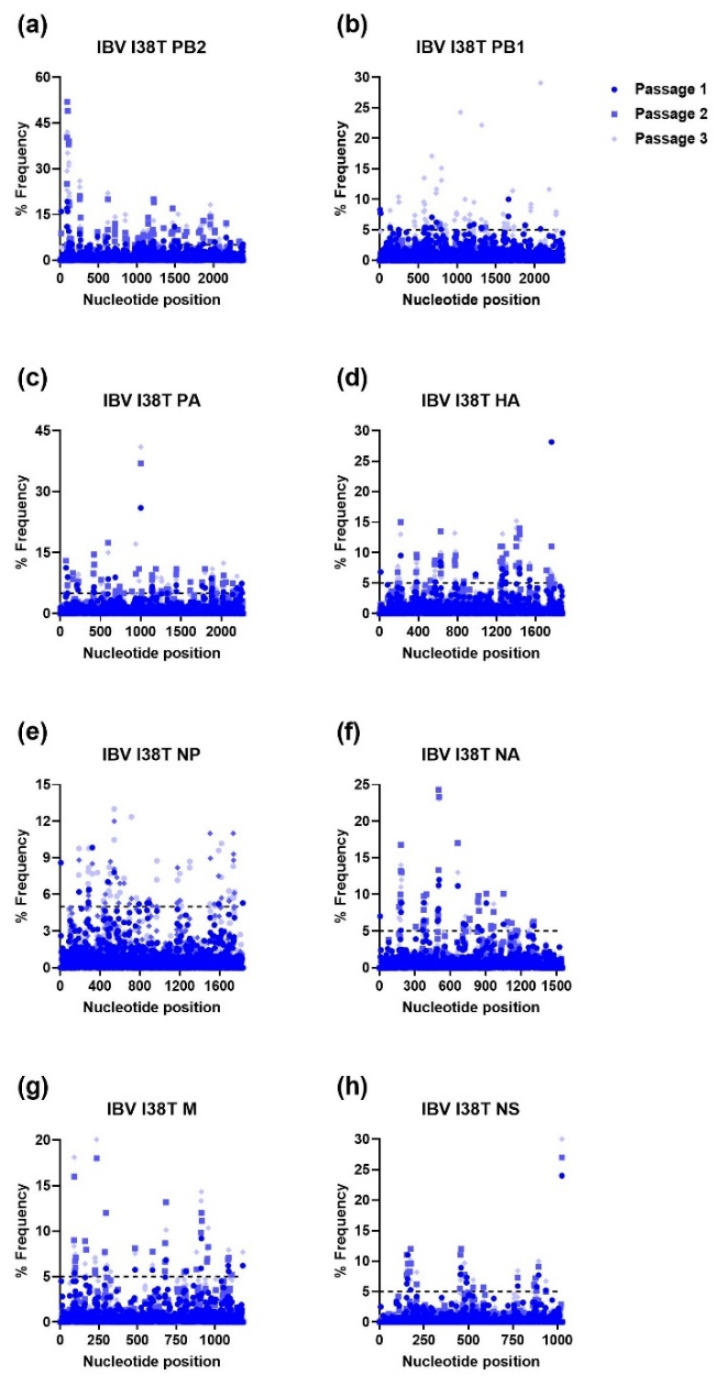
Percent frequency of mutations at each nucleotide loci for each passage within the IBV I38T genes ((**a**) PB2, (**b**) PB1, (**c**) PA, (**d**) HA, (**e**) NP, (**f**) NA, (**g**) M, (**h**) NS). To generate mutations, IBV I38T MUT virus was serially passaged three times in NHBE cells. NGS was then performed on RNA extracted from supernatant aliquots from each passage and the data was analyzed using the FDA in-house NGS data analysis platform, HIVE. The dashed line indicates the 5% cutoff mutation frequency. IBV, Influenza B virus; PB2, Polymerase basic 2; PB1, Polymerase basic 1; PA, Polymerase acidic; HA, Hemagglutinin; NP, Nucleoprotein; NA, Neuraminidase; M, Matrix; NS, Nonstructural.

For IBV WT, each passage resulted in numerous mutations in every viral gene with nucleotide loci mutation frequencies ranging from 20% to 56% (PB2; passage 1, Figure 7). A similar trend was observed for IBV I38T MUT, where the highest nucleotide loci mutation frequencies were observed in PB2 (52%) and every gene had nucleotide loci mutation frequencies of ≥20%, except for the nucleoprotein (NP) gene (Figure 8).

### 3.2. Ratio of Nonsynonymous to Synonymous Nucleotide Mutations and Comparison of Mutational Profiles between WT and MUT Genes after Each Passage

We next determined the total and nonsynonymous mutations in each viral gene after each passage and the d_N_/d_S_ ratios. For IAV H1N1, all of the mutations that occurred during serial passage were nonsynonymous, except for the single mutation that occurred after the first passage in the NS gene of the I38T MUT virus (Table 1). Notably, all of the IAV H1N1 viruses developed nonsynonymous mutations in the PB2 gene after the first passage. The other nonsynonymous mutations developed by the IAV H1N1s included a single mutation in the PB1 gene in the E199D MUT and single mutations in the PA gene of the WT and I38L MUT. The d_N_/d_S_ ratios for the IAV H1N1 viruses could not be calculated because either the synonymous or nonsynonymous mutations were 0. The mutational profiles of the I38L MUT NA after the third passage, the E199D MUT HA and NP after the second passage, and the E199D MUT NA genes after the third passage were significantly different compared to the respective WT virus. No significant differences in the mutation profiles were observed for the rest of the viral genes after each passage.

Passage of the IAV H3N2 WT led to nonsynonymous mutations in most genes for at least one passage (Table 1). The highest number of mutations was seen in the PB2 gene after the second passage. Most of the d_N_/d_S_ ratios for the IAV H3N2 WT were ≥1, with the exception being the second passage of the PB2 gene and the third passage of the PB1 gene, which had d_N_/d_S_ ratios of 0.9 and 0.6, respectively. We observed more mutations in every gene of the I38T MUT as compared to IAV H3N2 WT (Table 1 and Table S1). The largest number of mutations in each gene was observed after the first passage, which decreased after each subsequent passage. The majority of IAV H3N2 mutations induced AA substitutions resulting in d_N_/d_S_ ratios > 1 for most genes. Comparison of the mutation profiles between the IAV H3N2 WT and I38T MUT showed that over half (16/24) of the passages resulted in significantly different mutational profiles for most genes.

Serial passage of the IBV WT and MUT led to at least 11 mutations with >5% in frequency for every gene after every passage (Table 1). Furthermore, a large majority of the mutations that arose during serial passage were nonsynonymous and resulted in d_N_/d_S_ ratios of at least 2.5 or greater. Notably, the IBV WT NA gene had a d_N_/d_S_ ratio of 22.5 and the I38T MUT HA gene had a d_N_/d_S_ ratio of 20.0 after the first and second passages, respectively, which were the highest d_N_/d_S_ ratios of the serially passaged viruses used in this study. Significantly different mutational profiles were observed between the IBV WT and I38T MUT viruses after the first passage in PB2 and matrix (M), after the first and second passages in PB1, and after the third passage in NS. Collectively, serial passaging of the IAV H3N2 and IBV viruses resulted in most d_N_/d_S_ ratios being >1.0 (Table 1 and Table S1).

### 3.3. Mapping of AA Substitutions Occurred during Serial Passaging

**Figure 9:**
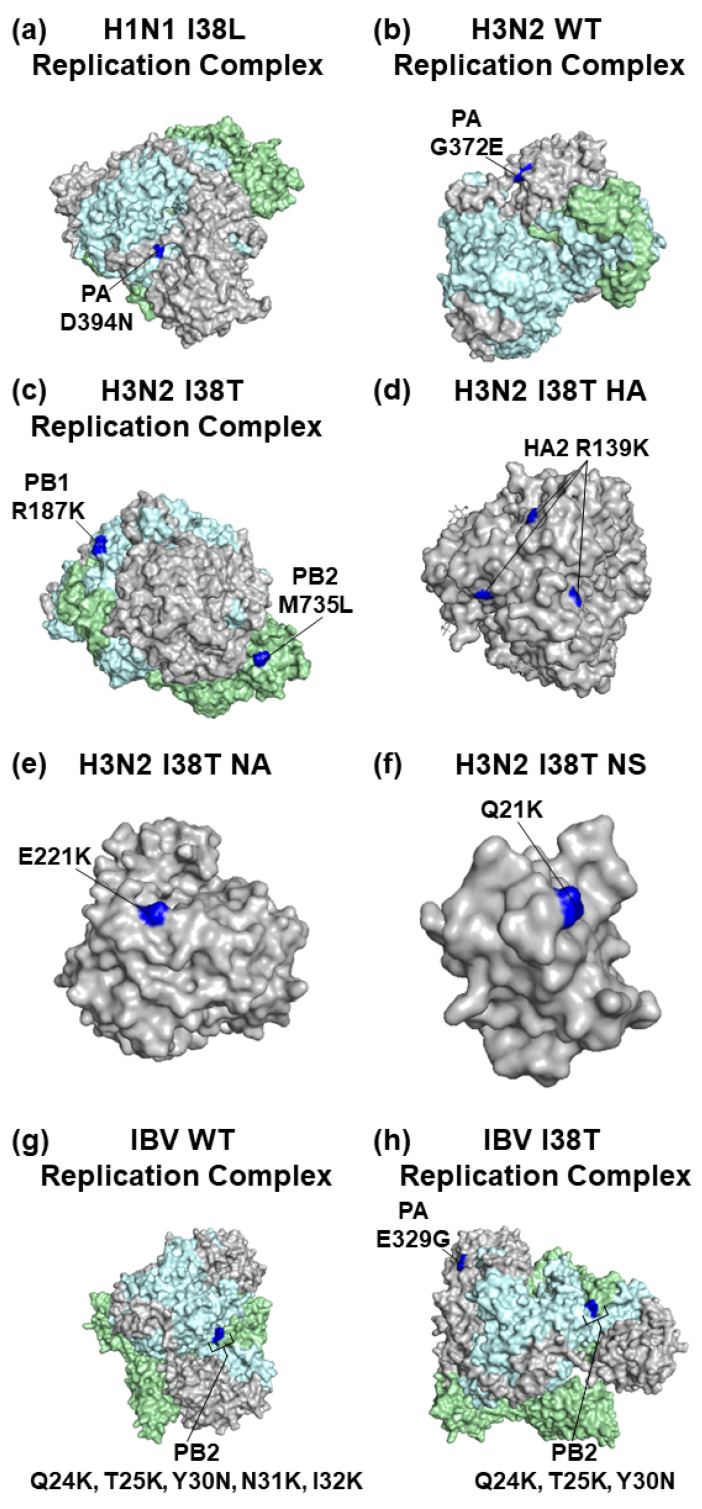
Location of substitutions in the influenza virus proteins that arose to frequencies of ≥30% after serial passage ((**a**) PB2, (**b**) PB1, (**c**) PA, (**d**) HA, (**e**) NP, (**f**) NA, (**g**) M, (**h**) NS). The locations of the substitutions are shown in blue and the substitution label is bolded. For the influenza virus replication centers, PB1 is colored light blue, PB2 is colored light green, and PA is colored gray. PA, Polymerase acidic; PB2, Polymerase basic 2; PB1, Polymerase basic 1; NS, Nonstructural; HA, Hemagglutinin; NA, Neuraminidase; RdRp, RNA-dependent RNA polymerase.

To gain additional insight into the changes that the influenza viruses underwent during serial passaging, AA substitutions that occurred at frequencies of ≥30% were identified and mapped on representative 3D influenza virus protein structures (Table 2 and Figure 9). We observed that only the PA D394N substitution occurred in the I38L MUT virus at a frequency of >30% during serial passaging (Table 2 and Figure 9a). While the PA G372E substitution occurred at a frequency of > 30% in the H3N2 WT virus (Figure 9b and Table 2), serial passage of the H3N2 I38T MUT virus resulted in AA substitutions in the PB2, PB1, HA, NA, and NS proteins (Figure 9c–f and Table 2). These substitutions occurred after the first passage in NHBE cells but dropped below 30% frequency after the second passage.

Passaged IBV WT developed 2 clusters of substitutions in the PB2 protein in residues 22–25 and 30–32 (Figure 9g and Table 2), including the Q24K and T25K substitutions that appeared after the first passage and remained until the end of the study at frequencies of >30%. Similarly, the serial passaged IBV I38T MUT developed several PB2 substitutions at the residues 24–30 (Figure 9h and Table 2) after the second passage. In addition, the IBV I38T MUT developed the PA E329G substitution after the second and third passages. Taken together, the IAV H1N1 and H3N2 WT viruses developed different AA substitutions than their MUT counterparts, but similar substitutions were observed in the IBV WT and MUT viruses.

## 4. Discussion

Understanding how influenza viruses carrying baloxavir resistance-associated substitutions evolve may aid in public health measures against these pathogens. Here, we used NGS to identify synonymous and nonsynonymous nucleotide mutations that arose in IAV H1N1 and H3N2, and IBV WTs and MUTs carrying baloxavir resistance-associated substitutions during serial passaging in NHBE cells. We then compared the resulting genomic mutational profiles of the MUT viruses to their respective WTs to determine if the presence of the drug resistance-associated substitutions impacted viral mutations and determined the d_N_/d_S_ ratios for each viral gene after each passage. We also mapped the AA substitutions that occurred at frequencies of > 30% on representative 3D structures for the respective influenza virus proteins.

The low-fidelity influenza virus polymerase is responsible for the development of nucleotide mutations in the viral genome and is a driving factor for the rapid evolution of influenza virus and antigenic “drift” [37]. In our study, we observed that the IAV H1N1 WT and MUTs were the most stable in terms of the development of mutations in the viral genome, followed by IAV H3N2 WT, and then by H3N2 I38T MUT, IBV WT, and I38T MUT. Our results are in contrast with those from Nobusawa and Sato [38], who reported that A/Aichi/1/87 (H1N1) and A/Aichi/12/92 (H3N2) demonstrated higher mutational rates compared to B/Aichi/29/99 and B/Aichi/44/01 viruses after passaging in MDCK cells. The reason for this discrepancy may be due to the different viral backgrounds and cell types used for passaging, indicating that the evolutionary rates of influenza viruses in cell culture are likely highly dependent on genetic background, cell type, and other culture conditions. In addition, the increase in the frequency of mutations within the genomes of the H3N2 and IBV I38T MUTs compared to their respective WTs indicates that the presence of I38T may have impacted IAV H3N2 and IBV viral replication capacities. Indeed, in our previous studies, we observed that while the IAV H1N1 virus carrying the PA I38T substitution did not have significantly altered replication kinetics compared to the respective WT virus, IBV I38T MUT replicated to a lower titer compared to IBV WT [13,26]. Further work is needed to determine the impact of PA I38T substitution in different viral backgrounds.

A common tool used to gain insight into the type and strength of selection occurring in influenza virus populations is the d_N_/d_S_ ratio [39]. Generally, a d_N_/d_S_ ratio of <1 indicates purifying selection, equal to 1 indicates neutral selection, and >1 indicates positive selection [39]. Our finding that most of the d_N_/d_S_ ratios for the IAV H3N2s and IBVs ranged from 2.0 to 22.5 indicates that these viruses may have been undergoing adaptive evolution during passage in NHBE cells. It has been previously shown that influenza HA and NA typically exhibit the highest mutation rates and have the largest d_N_/d_S_ ratios of the viral genes in the circulating influenza virus population and after adaption in vitro and in vivo [40]. Our finding that some of the highest d_N_/d_S_ ratios were observed in the IBV WT and I38T MUT HA and NA genes suggest that there was a strong positive selective pressure on IBV, perhaps related to optimizing attachment to and release from the target NHBE cells [41].

Our analysis of the AA substitutions that occurred at frequencies of >30% showed that the H3N2 I38T MUT virus developed PB2 M735L, PB1 R187K, HA2 R139K, NA E221K, and NS Q21K substitutions. The impact of the observed substitutions on the structure–function of the affected proteins is unknown and more studies are needed. The PB1 R187K substitution is located near the PB2 mRNA cap-binding site and has been previously reported to enhance replication efficacy together with other substitutions for A/duck/Nanjing/06/2003 (NJ06) and A/duck/Nanjing/01/1999 (NJ01) H9N2 viruses [42]. The NA E221K substitution located near the sialic acid binding site was observed in seasonal influenza A(H3N2) strains and arose in egg- and MDCK-cultured IAV H3N2 isolates Bris05AFN2 and Bris05MDN2 [43,44]. Further, both IBV WT and I38T MUT developed a cluster of PB2 substitutions at residues 24–32 residing within the loop-like region near the PB1 binding site and overlaps with the mitochondrial antiviral signaling protein (MAVS)-binding region [45], which plays an important role in inhibiting the antiviral interferon response. Further work is needed to assess the impact of the AA substitutions observed in serial passaged IBV WT and I38T MUT viruses on viral replication and interferon induction. To determine if the observed AA substitutions may also occur in the circulating influenza viruses, we analyzed influenza sequences deposited to the Influenza Virus Resource Database (https://www.ncbi.nlm.nih.gov/genomes/FLU/Database/nph-select.cgi?go=database (accessed on 29 September 2023) from 2018 to 2023. The IAV H1N1 PA D394N, IAV H3N2 PB1 R187K, NA E221K, NS Q21K, and IBV PA E329G substitutions were seen in the surveyed influenza virus population at frequencies of <1%).

Substitutions that enable antiviral resistance may come at a cost to the replication capacity of the virus [26,46,47]. Viruses carrying drug-resistant substitutions have been observed to develop concomitant substitutions that may help offset some of the replication capacity deficits conferred by the drug resistance-associated substitution [48,49,50]. In this study, we observed that the serial passage of the IAV H1N1 I38L MUT and the IBV I38T MUT resulted in the development of the PA D394N and E329G substitutions, respectively, located in the PA-arch of the C-terminal end in close proximity to PB1 [51]. We previously reported that serially passaged influenza A/California/04/09 (H1N1) virus with the PA I38L substitution in Calu-3 and NHBE cells and B/Victoria/504/2000 virus with the PA I38T in NHBE cells developed the same PA D394N and E329G substitutions, respectively [13,26]. We also showed that A/California/04/09 (H1N1) virus with the PA I38L and D394N substitutions had similar susceptibility to baloxavir inhibition as the parental IAV H1N1 PA I38L virus [26]. While the same virus stocks were used for all three studies, the serial passaging experiments were performed independently in different studies. Thus, we can speculate that the development of these additional PA substitutions may have compensated for replication changes reported in IAV H1N1 and IBV carrying PA I38L and PA I38T, respectively [13,24,26,52].

## 5. Conclusions

In conclusion, our data further our understanding of the impact of baloxavir resistance-associated substitutions on influenza virus mutation patterns and aid in the identification of substitutions that may impact viral properties. To our knowledge, our study is the first study where the mutation profiles of influenza viruses carrying baloxavir resistance-associated substitutions have been assessed after serial passaging. Since the emergence of baloxavir resistance in influenza virus populations threatens the continued effectiveness of this drug, understanding how resistant viruses evolve may aid in our preparedness for the newly emerged strains.

## Figures and Tables

**Table 1:** Ratios of nonsynonymous to total/synonymous nucleotide mutations and comparison of mutation profiles between WT and MUT virus genes after each passage.

Gene	Passage	H1N1				H3N2		IBV	
		WT	I38L	I38T	E199D	WT	I38T	WT	I38T
PB2	1	2/2 * (– ^†^)	2/2 (–)	1/1 (–)	1/1 (–)	0/0 (–)	**87/100 (6.7)**	95/104 (10.6)	**28/31 (9.3)**
	2	1/1 (–)	0/0 (–)	1/1 (–)	1/1 (–)	6/13 (0.9)	**33/43 (3.3)**	71/80 (7.9)	71/78 (10.1)
	3	0/0 (–)	0/0 (–)	0/0 (–)	1/1 (–)	0/1 (–)	6/12 (1.0)	67/74 (9.6)	65/72 (9.3)
PB1	1	0/0 (–)	0/0 (–)	0/0 (–)	1/1 (–)	1/2 (1.0)	**79/102 (3.4)**	79/98 (4.2)	**13/16 (4.3)**
	2	0/0 (–)	0/0 (–)	0/0 (–)	0/0 (–)	0/1 (–)	**32/41 (3.6)**	49/57 (6.1)	**53/65 (4.4)**
	3	0/0 (–)	0/0 (–)	0/0 (–)	0/0 (–)	3/8 (0.6)	5/6 (5.0)	56/65 (6.2)	54/63 (6.0)
PA	1	0/0 (–)	1/1 (–)	0/0 (–)	1/1 (–)	0/0 (–)	**81/98 (4.8)**	69/82 (5.3)	25/27 (12.5)
	2	0/0 (–)	1/1 (–)	0/0 (–)	0/0 (–)	10/13 (3.3)	**26/34 (3.3)**	55/60 (11)	49/54 (9.8)
	3	1/1 (–)	1/1 (–)	0/0 (–)	0/0 (–)	2/3 (2.0)	1/1 (–)	49/53 (12.3)	47/51 (11.8)
HA	1	0/0 (–)	0/0 (–)	0/0 (–)	0/0 (–)	0/0 (–)	**72/87 (4.8)**	54/60 (9.0)	11/13 (5.5)
	2	0/0 (–)	0/0 (–)	0/0 (–)	**0/0 (–)**	3/3 (–)	**35/44 (3.9)**	39/41 (19.5)	40/42 (20.0)
	3	0/0 (–)	0/0 (–)	0/0 (–)	0/0 (–)	0/0 (–)	**3/3 (N/A)**	38/41 (12.7)	42/45 (14.0)
NP	1	0/0 (–)	0/0 (–)	0/0 (–)	0/0 (–)	0/0 (–)	**45/60 (3.0)**	44/56 (3.7)	8/11 (2.7)
	2	0/0 (–)	0/0 (–)	0/0 (–)	**0/0 (–)**	4/6 (2.0)	**21/27 (3.5)**	23/32 (2.6)	29/37 (3.6)
	3	0/0 (–)	0/0 (–)	0/0 (–)	0/0 (–)	0/0 (–)	1/1 (–)	28/35 (4)	30/39 (3.3)
NA	1	0/0 (–)	0/0 (–)	0/0 (–)	0/0 (–)	1/1 (–)	**39/47 (4.9)**	45/47 (22.5)	21/23 (10.5)
	2	0/0 (–)	0/0 (–)	0/0 (–)	0/0 (–)	5/6 (5.0)	**26/33 (3.7)**	34/38 (8.5)	36/45 (4.0)
	3	0/0 (–)	**0/0 (–) ^‡^**	0/0 (–)	**0/0 (–)**	0/0 (–)	4/5 (4.0)	25/28 (8.3)	30/35 (6.0)
M	1	0/0 (–)	0/0 (–)	0/0 (–)	0/0 (–)	0/1 (–)	16/24 (2.0)	36/44 (4.5)	**10/14 (2.5)**
	2	0/0 (–)	0/0 (–)	0/0 (–)	0/0 (–)	7/8 (7.0)	**10/13 (3.3)**	23/30 (3.3)	30/35 (6.0)
	3	0/0 (–)	0/0 (–)	0/0 (–)	0/0 (–)	0/1 (–)	0/1 (–)	28/32 (7.0)	31/36 (6.2)
NS	1	0/0 (–)	0/0 (–)	0/1 (–)	0/0 (–)	0/0 (–)	**17/18 (17.0)**	55/63 (6.9)	10/14 (2.5)
	2	0/0 (–)	0/0 (–)	0/0 (–)	0/0 (–)	0/0 (–)	**10/14 (2.5)**	20/23 (6.7)	17/21 (4.3)
	3	0/0 (–)	0/0 (–)	0/0 (–)	0/0 (–)	0/0 (–)	1/1 (–)	17/18 (17.0)	**16/20 (4.0)**

* Ratio of nonsynonymous to total nucleotide mutations seen in the viral gene after the passage. ^†^ Ratio of nonsynonymous to synonymous mutations (d_N_/d_S_). ^‡^ Bold indicates statistically significant mutation profile observed in the MUT viral gene compared to the respective WT virus gene after the passage. –: Not applicable, because no nonsynonymous and/or synonymous nucleotide mutations were observed in the viral gene at frequencies over 5% after the passage.

**Table 2:** Number and location of amino acid substitutions observed during serial passaging at frequencies ≥ 5% or ≥30%.

Gene	Passage	H1N1								H3N2				IBV			
		WT		I38L		I38T		E199D		WT		I38T		WT		I38T	
		5%	30%	5%	30%	5%	30%	5%	30%	5%	30%	5%	30%	5%	30%	5%	30%
PB2	1	2 *	– ^‡^	2	–	1	–	1	–	0	–	87	M735L	95	Q24K ^§^, T25K, Y30N, N31K, I32K	28	–
	2	1	–	0	–	1	–	1	–	6	–	33	–	71	Q24K, T25K	71	Q24K, T25K, Y30N
	3	0	–	0	–	0	–	1	–	0	–	6	–	67	Q24K, T25K, Y30N	65	Q24K, T25K, Y30N
PB1	1	0	–	0	–	0	–	1	–	1	–	79	R187K	79	–	13	–
	2	0	–	0	–	0	–	0	–	0	–	32	–	49	–	53	–
	3	0	–	0	–	0	–	0	–	3	–	5	–	56	–	54	–
PA	1	0	–	1	–	0	–	1	–	0	–	81	–	69	–	25	–
	2	0	–	1	–	0	–	0	–	10	G372E	26	–	55	–	49	E329G
	3	1	–	1	D394N ^†^	0	–	0	–	2	G372E	1	–	49	–	47	E329G
HA	1	0	–	0	–	0	–	1	–	0	–	72	R139K	54	–	11	–
	2	0	–	0	–	0	–	0	–	3	–	35	–	39	–	40	–
	3	0	–	0	–	0	–	0	–	0	–	3	–	38	–	42	–
NP	1	0	–	0	–	0	–	0	–	0	–	45	–	44	–	8	–
	2	0	–	0	–	0	–	0	–	4	–	21	–	23	–	29	–
	3	0	–	0	–	0	–	0	–	0	–	1	–	28	–	30	–
NA	1	0	–	0	–	0	–	0	–	1	–	39	E221K	45	–	21	–
	2	0	–	0	–	0	–	0	–	5	–	26	–	34	–	36	–
	3	0	–	0	–	0	–	0	–	0	–	4	–	25	–	30	–
M	1	0	–	0	–	0	–	0	–	0	–	16	–	36	–	10	–
	2	0	–	0	–	0	–	0	–	7	–	10	–	23	–	30	–
	3	0	–	0	–	0	–	0	–	0	–	0	–	28	–	31	–
NS	1	0	–	0	–	0	–	0	–	0	–	17	Q21K	55	–	10	–
	2	0	–	0	–	0	–	0	–	0	–	10	–	20	–	17	–
	3	0	–	0	–	0	–	0	–	0	–	1	–	17	–	16	–

* Number of amino acid substitutions observed after each passage (nonsense mutations are not shown/all substitutions observed over 5% frequency are listed in Supplementary Materials File). ^†^ Amino acid substitution(s) occurred at frequencies ≥30%. ^‡^ No substitutions at frequencies ≥30% were observed. ^§^ Common substitutions developed by both WT and MUT viruses are underlined.

## Data Availability

All primary data leading to the presented figures and tables are available from the authors’ laboratory. The article reflects the views of the authors and should not be construed to represent FDA’s views or policies.

## Supplementary Materials

The following supporting information can be downloaded at: https://www.mdpi.com/article/10.3390/v15122446/s1, Table S1: Substitutions that arose with more than 5% frequency after each passage.

## References

[1] Krammer F., Smith G.J.D., Fouchier R.A.M., Peiris M., Kedzierska K., Doherty P.C., Palese P., Shaw M.L., Treanor J., Webster R.G. (2018). Influenza. Nat. Rev. Dis. Primers.

[2] Molinari N.A., Ortega-Sanchez I.R., Messonnier M.L., Thompson W.W., Wortley P.M., Weintraub E., Bridges C.B. (2007). The annual impact of seasonal influenza in the US: Measuring disease burden and costs. Vaccine.

[3] Dawood F.S., Iuliano A.D., Reed C., Meltzer M.I., Shay D.K., Cheng P.Y., Bandaranayake D., Breiman R.F., Brooks W.A., Buchy P. (2012). Estimated global mortality associated with the first 12 months of 2009 pandemic influenza A H1N1 virus circulation: A modelling study. Lancet Infect Dis..

[4] Girard M.P., Tam J.S., Assossou O.M., Kieny M.P. (2010). The 2009 A (H1N1) influenza virus pandemic: A review. Vaccine.

[5] Swierczynska M., Mirowska-Guzel D.M., Pindelska E. (2022). Antiviral Drugs in Influenza. Int. J. Environ. Res. Public Health.

[6] Hussain M., Galvin H.D., Haw T.Y., Nutsford A.N., Husain M. (2017). Drug resistance in influenza A virus: The epidemiology and management. Infect. Drug Resist..

[7] Petrova V.N., Russell C.A. (2018). The evolution of seasonal influenza viruses. Nat. Rev. Microbiol..

[8] Steinhauer D.A., Domingo E., Holland J.J. (1992). Lack of evidence for proofreading mechanisms associated with an RNA virus polymerase. Gene.

[9] Vahey M.D., Fletcher D.A. (2019). Low-Fidelity Assembly of Influenza A Virus Promotes Escape from Host Cells. Cell.

[10] Smyk J.M., Szydlowska N., Szulc W., Majewska A. (2022). Evolution of Influenza Viruses-Drug Resistance, Treatment Options, and Prospects. Int. J. Mol. Sci..

[11] Hurt A.C., Holien J.K., Parker M., Kelso A., Barr I.G. (2009). Zanamivir-resistant influenza viruses with a novel neuraminidase mutation. J. Virol..

[12] Ince W.L., Smith F.B., O’Rear J.J., Thomson M. (2020). Treatment-Emergent Influenza Virus Polymerase Acidic Substitutions Independent of Those at I38 Associated with Reduced Baloxavir Susceptibility and Virus Rebound in Trials of Baloxavir Marboxil. J. Infect. Dis..

[13] Hickerson B.T., Petrovskaya S.N., Dickensheets H., Donnelly R.P., Ince W.L., Ilyushina N.A. (2023). Impact of Baloxavir Resistance-Associated Substitutions on Influenza Virus Growth and Drug Susceptibility. J. Virol..

[14] Hickerson B.T., Adams S.E., Bovin N.V., Donnelly R.P., Ilyushina N.A. (2022). Generation and characterization of interferon-beta-resistant H1N1 influenza A virus. Acta Virol..

[15] Omoto S., Speranzini V., Hashimoto T., Noshi T., Yamaguchi H., Kawai M., Kawaguchi K., Uehara T., Shishido T., Naito A. (2018). Characterization of influenza virus variants induced by treatment with the endonuclease inhibitor baloxavir marboxil. Sci. Rep..

[16] Noshi T., Kitano M., Taniguchi K., Yamamoto A., Omoto S., Baba K., Hashimoto T., Ishida K., Kushima Y., Hattori K. (2018). In vitro characterization of baloxavir acid, a first-in-class cap-dependent endonuclease inhibitor of the influenza virus polymerase PA subunit. Antivir. Res..

[17] Abed Y., Baz M., Boivin G. (2006). Impact of neuraminidase mutations conferring influenza resistance to neuraminidase inhibitors in the N1 and N2 genetic backgrounds. Antivir. Ther..

[18] Suzuki H., Saito R., Masuda H., Oshitani H., Sato M., Sato I. (2003). Emergence of amantadine-resistant influenza A viruses: Epidemiological study. J. Infect. Chemother..

[19] Dharan N.J., Gubareva L.V., Meyer J.J., Okomo-Adhiambo M., McClinton R.C., Marshall S.A., St George K., Epperson S., Brammer L., Klimov A.I. (2009). Infections with oseltamivir-resistant influenza A(H1N1) virus in the United States. JAMA.

[20] Okomo-Adhiambo M., Sleeman K., Ballenger K., Nguyen H.T., Mishin V.P., Sheu T.G., Smagala J., Li Y., Klimov A.I., Gubareva L.V. (2010). Neuraminidase inhibitor susceptibility testing in human influenza viruses: A laboratory surveillance perspective. Viruses.

[21] Hayden F.G., Sugaya N., Hirotsu N., Lee N., de Jong M.D., Hurt A.C., Ishida T., Sekino H., Yamada K., Portsmouth S. (2018). Baloxavir Marboxil for Uncomplicated Influenza in Adults and Adolescents. N. Engl. J. Med..

[22] Checkmahomed L., M’Hamdi Z., Carbonneau J., Venable M.C., Baz M., Abed Y., Boivin G. (2020). Impact of the Baloxavir-Resistant Polymerase Acid I38T Substitution on the Fitness of Contemporary Influenza A(H1N1)pdm09 and A(H3N2) Strains. J. Infect. Dis..

[23] Chesnokov A., Patel M.C., Mishin V.P., De La Cruz J.A., Lollis L., Nguyen H.T., Dugan V., Wentworth D.E., Gubareva L.V. (2020). Replicative Fitness of Seasonal Influenza A Viruses With Decreased Susceptibility to Baloxavir. J. Infect. Dis..

[24] Jones J.C., Pascua P.N.Q., Fabrizio T.P., Marathe B.M., Seiler P., Barman S., Webby R.J., Webster R.G., Govorkova E.A. (2020). Influenza A and B viruses with reduced baloxavir susceptibility display attenuated in vitro fitness but retain ferret transmissibility. Proc. Natl. Acad. Sci. USA.

[25] Koszalka P., Tilmanis D., Roe M., Vijaykrishna D., Hurt A.C. (2019). Baloxavir marboxil susceptibility of influenza viruses from the Asia-Pacific, 2012–2018. Antivir. Res..

[26] Hickerson B.T., Adams S.E., Barman S., Miller L., Lugovtsev V.Y., Webby R.J., Ince W.L., Donnelly R.P., Ilyushina N.A. (2022). Pleiotropic Effects of Influenza H1, H3, and B Baloxavir-Resistant Substitutions on Replication, Sensitivity to Baloxavir, and Interferon Expression. Antimicrob. Agents Chemother..

[27] Takizawa N., Momose F. (2022). A novel E198K substitution in the PA gene of influenza A virus with reduced susceptibility to baloxavir acid. Arch. Virol..

[28] Jones J.C., Zagribelnyy B., Pascua P.N.Q., Bezrukov D.S., Barman S., Okda F., Webby R.J., Ivanenkov Y.A., Govorkova E.A. (2022). Influenza A virus polymerase acidic protein E23G/K substitutions weaken key baloxavir drug-binding contacts with minimal impact on replication and transmission. PLoS Pathog..

[29] Jester B., Schwerzmann J., Mustaquim D., Aden T., Brammer L., Humes R., Shult P., Shahangian S., Gubareva L., Xu X. (2018). Mapping of the US Domestic Influenza Virologic Surveillance Landscape. Emerg. Infect. Dis..

[30] Van Poelvoorde L.A.E., Saelens X., Thomas I., Roosens N.H. (2020). Next-Generation Sequencing: An Eye-Opener for the Surveillance of Antiviral Resistance in Influenza. Trends Biotechnol..

[31] Roosenhoff R., Schutten M., Reed V., Clinch B., van der Linden A., Fouchier R.A.M., Fraaij P.L.A. (2021). Secondary substitutions in the hemagglutinin and neuraminidase genes associated with neuraminidase inhibitor resistance are rare in the Influenza Resistance Information Study (IRIS). Antivir. Res..

[32] Ilyushina N.A., Lugovtsev V.Y., Samsonova A.P., Sheikh F.G., Bovin N.V., Donnelly R.P. (2017). Generation and characterization of interferon-lambda 1-resistant H1N1 influenza A viruses. PLoS ONE.

[33] Matrosovich M.N., Matrosovich T.Y., Gray T., Roberts N.A., Klenk H.D. (2004). Human and avian influenza viruses target different cell types in cultures of human airway epithelium. Proc. Natl. Acad. Sci. USA.

[34] Hoffmann E., Neumann G., Kawaoka Y., Hobom G., Webster R.G. (2000). A DNA transfection system for generation of influenza A virus from eight plasmids. Proc. Natl. Acad. Sci. USA.

[35] Hoffmann E., Stech J., Guan Y., Webster R.G., Perez D.R. (2001). Universal primer set for the full-length amplification of all influenza A viruses. Arch. Virol..

[36] Simonyan V., Chumakov K., Dingerdissen H., Faison W., Goldweber S., Golikov A., Gulzar N., Karagiannis K., Vinh Nguyen Lam P., Maudru T. (2016). High-performance integrated virtual environment (HIVE): A robust infrastructure for next-generation sequence data analysis. Database.

[37] Barbezange C., Jones L., Blanc H., Isakov O., Celniker G., Enouf V., Shomron N., Vignuzzi M., van der Werf S. (2018). Seasonal Genetic Drift of Human Influenza A Virus Quasispecies Revealed by Deep Sequencing. Front. Microbiol..

[38] Nobusawa E., Sato K. (2006). Comparison of the mutation rates of human influenza A and B viruses. J. Virol..

[39] Jeffares D.C., Tomiczek B., Sojo V., dos Reis M. (2015). A beginners guide to estimating the non-synonymous to synonymous rate ratio of all protein-coding genes in a genome. Methods Mol. Biol..

[40] Bhatt S., Holmes E.C., Pybus O.G. (2011). The genomic rate of molecular adaptation of the human influenza A virus. Mol. Biol. Evol..

[41] Ilyushina N.A., Lee N., Lugovtsev V.Y., Kan A., Bovin N.V., Donnelly R.P. (2020). Adaptation of influenza B virus by serial passage in human airway epithelial cells. Virology.

[42] Liu Q., Liu Y., Yang J., Huang X., Han K., Zhao D., Bi K., Li Y. (2016). Two Genetically Similar H9N2 Influenza A Viruses Show Different Pathogenicity in Mice. Front. Microbiol..

[43] Kim J.I., Lee I., Park S., Bae J.Y., Yoo K., Cheong H.J., Noh J.Y., Hong K.W., Lemey P., Vrancken B. (2017). Phylogenetic relationships of the HA and NA genes between vaccine and seasonal influenza A(H3N2) strains in Korea. PLoS ONE.

[44] Mohr P.G., Deng Y.M., McKimm-Breschkin J.L. (2015). The neuraminidases of MDCK grown human influenza A(H3N2) viruses isolated since 1994 can demonstrate receptor binding. Virol. J..

[45] Patel D., Schultz L.W., Umland T.C. (2013). Influenza A polymerase subunit PB2 possesses overlapping binding sites for polymerase subunit PB1 and human MAVS proteins. Virus Res..

[46] Ives J.A., Carr J.A., Mendel D.B., Tai C.Y., Lambkin R., Kelly L., Oxford J.S., Hayden F.G., Roberts N.A. (2002). The H274Y mutation in the influenza A/H1N1 neuraminidase active site following oseltamivir phosphate treatment leave virus severely compromised both in vitro and in vivo. Antivir. Res..

[47] Abed Y., Goyette N., Boivin G. (2004). A reverse genetics study of resistance to neuraminidase inhibitors in an influenza A/H1N1 virus. Antivir. Ther..

[48] Bloom J.D., Gong L.I., Baltimore D. (2010). Permissive secondary mutations enable the evolution of influenza oseltamivir resistance. Science.

[49] Hurt A.C., Hardie K., Wilson N.J., Deng Y.M., Osbourn M., Leang S.K., Lee R.T., Iannello P., Gehrig N., Shaw R. (2012). Characteristics of a widespread community cluster of H275Y oseltamivir-resistant A(H1N1)pdm09 influenza in Australia. J. Infect. Dis..

[50] Butler J., Hooper K.A., Petrie S., Lee R., Maurer-Stroh S., Reh L., Guarnaccia T., Baas C., Xue L., Vitesnik S. (2014). Estimating the fitness advantage conferred by permissive neuraminidase mutations in recent oseltamivir-resistant A(H1N1)pdm09 influenza viruses. PLoS Pathog..

[51] Pflug A., Guilligay D., Reich S., Cusack S. (2014). Structure of influenza A polymerase bound to the viral RNA promoter. Nature.

[52] Pascua P.N.Q., Jones J.C., Webby R.J., Govorkova E.A. (2022). Effect of E23G/K, F36V, N37T, E119D, and E199G polymerase acidic protein substitutions on the replication and baloxavir susceptibility of influenza B viruses. Antivir. Res..

