# Supplementary material for: Genomic Analysis of Influenza A and B Viruses Carrying Baloxavir Resistance-Associated Substitutions Serially Passaged in Human Epithelial Cells
Source: Viruses. 2023 Dec 16;15(12):2446. doi: 10.3390/v15122446 (PMC10748225; doi:10.3390/v15122446)
Supplement: Supplementary file 1 [file viruses-15-02446-s001.zip › viruses-2750757-supplementary.pdf]

Table S1: Substitutions that arose over 5% frequency after each passage

Protein	Passage	H1N1			
		**WT**	**I38L**	**I38T**	**E199D**
**PB2**	**1**	N71I	V734E	**K738N**	**K738N**
		**K738N**[*]	**K738N**		
	**2**	**K738N**	N/A	**K738N**	**K738N**
	**3**	N/A[†]	N/A	N/A	K738N
**PB1**	**1**	N/A	N/A	N/A	L753I
	**2**	N/A	N/A	N/A	N/A
	**3**	N/A	N/A	N/A	N/A
**PA**	**1**	N/A	D394N	N/A	E26K
	**2**	N/A	D394N	N/A	N/A
	**3**	I38L	D394N	N/A	N/A
**HA**	**1**	N/A	N/A	N/A	HA2 S215A
	**2**	N/A	N/A	N/A	N/A
	**3**	N/A	N/A	N/A	N/A
**HA2**	**1**	N/A	N/A	N/A	N/A
	**2**	N/A	N/A	N/A	N/A
	**3**	N/A	N/A	N/A	N/A
**NA**	**1**	N/A	N/A	N/A	N/A
	**2**	N/A	N/A	N/A	N/A
	**3**	N/A	N/A	N/A	N/A
**NP**	**1**	N/A	N/A	N/A	N/A
	**2**	N/A	N/A	N/A	N/A
	**3**	N/A	N/A	N/A	N/A
**M1**	**1**	N/A	N/A	N/A	N/A
	**2**	N/A	N/A	N/A	N/A
	**3**	N/A	N/A	N/A	N/A
**M2**	**1**	N/A	N/A	N/A	N/A
	**2**	N/A	N/A	N/A	N/A
	**3**	N/A	N/A	N/A	N/A
**NS**	**1**	N/A	N/A	N/A	N/A
	**2**	N/A	N/A	N/A	N/A
	**3**	N/A	N/A	N/A	N/A

[*]Bolded subsitutions indicate common substitutions between WT and MUT virus

[†]N/A: Not applicable; No substitutions were observed in the viral protein after passage

Substitutions that arose over 5% frequency after each passage

Protein	Passage	H3N2	
		**WT**	**I38T**
		R213G	T21K
		D309G	H27N
		D506G	I30K
		A569T	G37E
		Y592N	Y55C
		L733S	D60H
			E65K
			E69Stop
			R70K
			E72K
			Q73K
			G74E
			G75K
			S79N/R
			Y111N/Stop
			P112T/Q
			D120E
			E171K
			L177G
			E191K
			T215K
			L232F
			Q236H
			R299Stop
			D309N
			R318K
			F325I
			S336A
			V338I
			E343K
			L352S
			I354K
			E358Stop
			G359V
			G367R/E
			G388E
			I411L
	**1**		K412T
			A413T/G
			G459E
			M483L
			L502F
			D506Y
			N510I

			L512Stop
			S519R
			E520K
			N548K
			T549I
			Y550D
			W552R
			I553F
			Q563K
			W564L
			Y572Stop
**PB2**			E576K
			M645I
			R646K
			R664K
			T666K
			G669R/E
			P679Q
			E712K
			A717T/E
			L725I
			I726F/S
			Q728H
			G729V
			D730E/V
			M735I/L
			K736N/Stop
			R737L
			L738N
			D746H
			L20I/Q
			T21K
			T23P
			D26N
			H27N
			I30K
			G74E
			G75K
			P169S
			E188K
			E191K
			E192K
			P219Q
			V267E
			R268K
	**2**	N/A†	E305K
			Q306K

			T333K
			E342K
			E343K
			I354K
			T666K
			L675Stop
			N723I
			Q728K
			D730E/V
			V731G
			V734E
			M735I
			R737P/L
			L738N
	**3**	N/A	E305G/Stop
			G367R
			Q433H
			Y572F
			Q728K
		C770R	A140S
			A14E
			A349V
			A453E
			E109D
			E178K
			E256K
			E390K
			E60K
			F185V/C/L
			F412L
			G234D
			G513V/Stop
			G622E
			H634Q
			I18K
			I248N
			I392K
			I674F
			I750F
			K237N
			K578Stop
			L163Q
			L236I/Stop
			L550M
			L675R
			M246I
			M317L

PB1	1		M409I
			M546K
			N134K
			N16K
			N425I
			N58K
			N641K
			N671D
			N676K
			P394H
			P454T/Q
			P596Q
			Q186K/H
			Q247K
			Q268L
			Q313K
			Q367K
			Q460K
			Q48L
			R135K/Stop
			R187K
			R233K
			S173Stop
			S19R
			S633R
			T132A
			T20K
			T307A
			T366K
			T36A
			T432N
			T434K
			T543P
			T59K
			T677K
			V421L
			V640E
			W437Stop
			Y253N
			Y355C
			Y555Stop
			E277K
			E311K
			E383K
			E636K
			E78K
			F185L

	2	N/A	I181K
			I219K
			I342K
			I552N
			L218I/Q
			L271I
			L576Q
			N276K
			N312K
			N346K
			N536K
			P369H
			P596Q
			Q313K
			Q549K
			Q567K
			R187K
			R287G
			S384T/Stop
			T110K
			T20K
			T21K
			T385K
			T677K
	3	P369A	E256K
		S704Stop	G594R
		Y436Stop	I389T
			L258I
			S261R
		L42W	C7S
		E43Q	C10S
		D108N	N14K
		K134E	E15K
		P238L	G20E
		G372E	N25K
		E430V	Q27K
		I486R	Stop49R
		Y722H	T50K
			V65D
			Q77K
			N78K
			S84R
			Q106K
			Stop132K
			E135K
			G147E
			H171Q

PA	1		T173K
			R174K
			P180T
			R188Q
			E215Q
			P249L
			F253L
			D256V
			N261I/K
			Q262K
			N306T
			R320Q
			G322V
			F325Y/L
			M331I
			R337K
			A339S
			D350N
			W365Stop
			H380Q
			E384Stop
			R390Q
			L415M
			E435V
			H460N
			N484K
			Stop485K
			A489E
			I494L
			E524K
			Stop526L
			A527D
			T543N
			Y554Stop
			V557L
			N568K
			E569K
			Stop590W
			E598K
			Q602K
			R610K
			H614N
			C662F
			S676C
			L692P
			A695S
			L703P

			Y705Stop
			F717L
	2	N/A	C7S
			A20E
			M21K/I
			D27N
			N33K
			I178K
			R179K
			Q180K
			E181K
			E195K
			I268N
			Y277F
			G372E
			E377K
			L500F
			Y501F
			L550I/Q
			T567K
			S571Stop
			E604K
			Q654K
			V669F
			L701F
	3	N/A	C7S
	1	N/A	D7E
			H18N
			N22D
			V26E
			T28S/K
			A106V
			F120Y/L
			N121K
			G129Stop
			T131S
			A138S
			H156Q
			L157I/Stop
			L164Stop
			M168K/I
			P169T/Q
			N170K
			N171Y/K
			E172K
			H183Q
			G200V

HA1			T206N
			Q211L
			P215T
			N216D
			I217T
			Q327K/L
			A334E
			A336E
			G337C
			G342R
			W343L
	2	T135R	G5R/E
		P254H	D7E
		R261Stop	P169T/Q
			N170K
			N171K
			E172K
			T206N
			R208K
			I236K
			Q295E
			Q327K
	3	N/A	C100R
	1	N/A	M3I
			V4L
			G6C
			W7R
			Q20K
			D23H
			N32K
			Q33K
			N46H
			F49I
			E53D
			K54I
			E55K/V
			E60V
			G61V
			V70D
			T93K
			E114K
			F124L
			I138N
			R139K
			N140K
			H145N
			V147I

**HA2**			Q158K
			I159N
			S176F
			C185F
			V186F
			L188F
			L189F
	**2**	N/A	N31K
			Q32K
			G42E
			E46K
			R61K
			H91N/Q
			T92K
			R106K
			T107K
			E113K
			F123L
			R138K
			V146I
			I158N
			L182F
			C184F
			V185F
			A186S/G/V
			L187F
	**3**	N/A	K27R
			Y71H
			A131S
			A237E
			C333Stop
			D112N/E
			D72E
			E114K/Stop
			E115K/Stop
			E11D
			E14K/D
			E294K
			E454K
			E455K
			E64K
			E81K
			G356E
			G5S
			I116K
			I257L
	**1**	N/A	I347N

**NP**			I63K
			I96M
			K198R/N
			L143F
			M137I
			M13I
			N124K
			P283T
			P419Q
			Q235K
			Q308K
			R117T
			R31K
			S310G
			T62K
			Y111N/Stop
			Y289N
			Y52N
	**2**	A251T	M13I
		V280A	E14K/D
		N417D	G102V
			Y111F
			D112N/E
			E113K
			I116K
			R117T
			Y120R
			D128G
			R195K
			T215K
			R216K
			Q235K
			E244K
			G295E
			H324Q
			P419Q
			E454K
			E455K
	**3**	N/A	Y496H
			S14P
			L15H
			V33E
			P46Q
			Q49K
			E54K
			P55T/Q
			T56K

NA	1	N/A	I58K
			R60K
			E74K
			H168Y
			G196W
			A201S/E
			N208Y/I
			R210S
			L211H
			E221K
			S266C
			V303E
			D304N/E
			I305K
			R327T
			C337S
			E344K
			G357R/E
			E375K
			F377I
			Q395L
			R430K
			V445L
			C447F
			G448C
	2	N2I	S18F
		K80E	M24I
		W87R	Q25K
		E343Stop	S101F
		S367N	T138K
			L140I/Q
			N141K
			H168Y
			E221K
			C318G
			R327K
			E344K
			G363E
			R364K
			E375K
			I392K
			S411F
			V412F
			R430K
			E433K
			V445L
			C447F

			E476K/A/D
	3	N/A	L321I
			S332P
			R430G
			G461E
M1	1	N/A	E23G
			E44K
			T48K
			K57T
			I131K
			Y132N/Stop
			R210M
			Q238K
			Stop253S
			R259H
			Y261H
			W263L
			A266E
			L267I
			Q285H
	2	E6K	P50Q
		E152D	V63C
		M203K/L	Y132N/Stop
		D268V	V138E
		S275C	N177K
			R178K
			E201D
			Stop289K
			C313R
	3	N/A	
M2	1	E14V	D24H
		L46V	A30T
			G34W
			H37N/Q
			K56I
	2	N/A	
	3	N/A	
NS	1	N/A	Q21K
			D44G
			T48S
			E56K
			T57A
			G58C
			L80F
			S97Stop
			N102Y
			K110Stop

			N111Y
			Q113L/H
			W117C
			S119C
			N123Y/I
	**2**	N/A	Q21K
			V23I/E
			D24N
			L77I/H
			I90K
			N101I
			E112D
			D173N
	**3**	N/A	V111A

[*]Bolded substitutions indicate common substitutions between WT and mutant virus

[†]N/A: Not applicable; No substitutions were observed in the viral protein after passage

Substitutions that arose over 5% frequency after each passage

Protein	Passage	IBV	
		**WT**	**I38T**
	1	V21E	L22Stop
		**L22Stop**[*]	**Q24K**
		**Q24K**	**T25K**
		**T25K**	Y30Stop
		D28N/E	**I32K**
		Q29K	**I77K**
		Y30N/Stop	**Q78K**
		N31K	**T81K**
		**I32K**	**E155K**
		I33K	**E190K**
		R34K	**I200K**
		R40K	S276Stop
		L47Stop	L332Q
		M49I	**Q354K**
		L72F	**I388K**
		G76E	**M396K/I**
		**I77K**	**L399F/Stop**
		**Q78K**	**R443K/I**
		L79I/H	**S490C/I**
		**T81K**	T500K
		**E155K**	L505Stop
		**E190K**	**I506K**
		R192K	**I616K**
		E193K	**E666K**
		**I200K**	**E716K**
		F230Y/L	G733E
		I231K	
		E232K	
		R243K	
		S276T/Stop	
		E284K	
		R324T/I	
		Q325K	
		R334K	
		F340Y/L	
		**Q354K**	
		I375K	
		L376I/Stop	
		R382K	
		L387Q	
		**I388K**	
		D395E	
		**M396K/I**	
		**L399F/Stop**	
		I400K	

		L430Stop	
		Q437K	
		**R443K/I**	
		M469I	
		V480E	
		V481I/E	
		T482K	
		**S490C/I**	
		S491F	
		S504N/R	
		L505I/Stop	
		**I506K**	
		G541E	
		T542K	
		T543N	
		E545K	
		L546P	
		Q548K	
		Q552E	
		W553C	
		G568Stop	
		E570K	
		I584K	
		A597S	
		Q603K	
		**I616K**	
		G646R/E	
		E647K	
		T665K	
		**E666K**	
		**E716K**	
		L720I/Q	
		V738I	
		**L22M/Stop**	**A597S**
		**Q24K**	**D395E**
		**T25K**	**E155K**
		**Y30N/Stop**	**E190K**
		**N31K**	**E193K**
		**I32K**	**E232K**
		**I33K**	**E284K**
		**R34K**	**E647K**
		G76E	**E666K**
		**I77K**	**E716K**
		**Q78K**	**F230Y/L**
		**L79I/H**	**F340L**
		**T81K**	F489L
		**E155K**	**F615L**
		**E190K**	G646R/E
		**R192K**	**G733E**

**PB2**		**E193K**	**I200K**
		**M199I**	**I231K**
		**I200K**	**I32K**
		**F230Y/L**	**I33K**
		**I231K**	**I375K**
		**E232K**	**I388K**
		**S276T/Stop**	**I400K**
		V283E	**I506K**
		**E284K**	**I616K**
		**L319Q**	**I77K**
		**L332I/Q**	**L22M/Stop**
		**R334K**	**L319Q**
	**2**	**F340L**	**L332I/Q**
		**Q354K**	**L376I/Stop**
		**I375K**	**L387I/Q**
		**L376I/Stop**	**L399F/Stop**
		**L387I/Q**	**L505Stop**
		**I388K**	**L79I/H**
		**D395E**	**M199I**
		**M396K/I**	**M396K/I**
		**L399F/Stop**	**N31K**
		**I400K**	**Q24K**
		Q437K	**Q354K**
		**R443K/I**	**Q603K**
		**V481I/E**	**Q78K**
		**T482K**	**R192K**
		S490C/I	**R334K**
		**L505Stop**	**R34K**
		**I506K**	R40K
		**T542K**	**R443K/I**
		T543N	**S276T/Stop**
		E545K	S407F
		**A597S**	S490C
		**Q603K**	**T25K**
		**F615L**	**T482K**
		**I616K**	**T542K**
		G646E	**T665K**
		**E647K**	**T81K**
		**T665K**	**V481I/E**
		**E666K**	**Y30N/Stop**
		**E716K**	Y772N/Stop
		**G733E**	
		L22Stop	**A597S**
		**Q24K**	**D395E**
		**T25K**	D683E
		**Y30N/Stop**	**E155K**
		**N31K**	**E190K**
		**I32K**	**E232K**
		**I33K**	**E284K**

		**R34K**	E545K
		R40K	**E647K**
		**I77K**	**E666K**
		**Q78K**	E684K
		**L79I/H**	E685K
		**T81K**	**E716K**
		**E155K**	**F230Y/L**
		**E190K**	**F340L**
		R192K	**F615L**
		E193K	**G646R/E**
		**M199I**	G733E
		**I200K**	**I200K**
		**F230Y/L**	**I231K**
		**I231K**	**I32K**
		**E232K**	**I33K**
		**S276T/Stop**	**I375K**
		V283E	**I388K**
		**E284K**	**I400K**
		L319Q/I	I499K
		**L332I/Q**	**I506K**
		**R334K**	**I616K**
	**3**	**F340L**	**I77K**
		**Q354K**	L22M/Stop
		**I375K**	**L332I/Q**
		**L376I/Stop**	**L376I/Stop**
		L387I/Q	**L399F/Stop**
		**I388K**	**L505Stop**
		**D395E**	**L79I/H**
		**M396K/I**	**M199I**
		**L399F/Stop**	**M396K/I**
		**I400K**	**N31K**
		**R443K/I**	**Q24K**
		**V481I/E**	**Q354K**
		**T482K**	**Q603K**
		F489L	**Q78K**
		**S490C**	**R334K**
		**T500K**	**R34K**
		**L505Stop**	**R443K/I**
		**I506K**	**S276T/Stop**
		**T542K**	**S490C**
		**T543N**	**T25K**
		**A597S**	**T482K**
		**Q603K**	**T500K**
		**F615L**	**T542K**
		**I616K**	**T543N**
		**G646R/E**	**T665K**
		**E647K**	**T81K**
		**T665K**	**V481I/E**
		**E666K**	**Y30N/Stop**

		**E716K**	
	1	A140P	**E399K**
		A259D/T	**G431E**
		A387E/S	I187K
		A452E	I218N
		A543E	I547K
		A744D	N432K
		C469Y	Q548K
		C560F	R196K
		C63Stop	**R238K**
		D175N/E	**T39K**
		D553V	Y217Stop
		D76N/E	Y381Stop
		E178K/D	Y619Stop
		E383K/V	
		E384K	
		**E399K**	
		E618K	
		E696Stop	
		E740Stop	
		E748Stop	
		E75K	
		**G431E**	
		G525E	
		G53E	
		H38Q	
		H745L	
		I201K	
		I357K	
		K185Stop	
		K188Stop	
		K347N	
		K360I	
		K362I/Stop	
		L404F	
		M356I/K	
		N186I	
		N432H/K	
		N535K	
		N581K	
		P138H	
		Q168K	
		Q367K	
		Q544K	
		Q548H/K	
		Q686K	
		R203K	
		**R238K**	
		R316K	

PB1		R380S	
		R555M	
		R733I	
		S291G	
		S359N/R	
		Stop753L/K	
		T301R	
		T358K	
		T361K	
		T385K	
		**T39K**	
		T492K	
		T64K	
		T667N	
		T69K	
		V252L	
		V450F	
		Y619N/Stop	
	2	**H38Q**	**A259T/D**
		**T39K**	A543E
		**E75K**	**D616N**
		**D76N/E**	D643N/E
		L143Stop	**D76N/E**
		**T145K**	E277K
		**V184I/E**	**E399K**
		**N186K**	**E75K**
		**I187K**	G431E
		R196K	**G525E**
		**R203K**	G53E
		V208E	**H38Q**
		**I218N**	**I187K**
		**L236Q**	**I218N**
		**R238K**	**I325K**
		**V252L**	**I427N**
		**A259T/D**	**I528K**
		**R316K**	**I529K**
		**R324K**	**I644K**
		**I325K**	L143I/Stop
		**S359N/R**	**L236Q**
		**T361K**	L779P
		**N397K**	**N186K**
		E398K	**N397K**
		**E399K**	**N432K**
		**I427N**	N51K
		**N432K**	Q104K
		L436Stop	Q544K
		I474K	**Q548K**
		E485K	**Q686K**
		**G525E**	**R203K**

	T527K	**R238K**
	**I528K**	**R316K**
	**I529K**	**R324K**
	**Q548K**	**R722K**
	S565Y	**S359N/R**
	G569R/E	**T145K**
	R571K	**T361K**
	**D616N**	**T39K**
	D643E	T64K
	**I644K**	**V184I/E**
	**Q686K**	**V252L**
	**R722K**	V450F
	T752K	Y217Stop
	Stop753K	Y381Stop
		Y619N/Stop
**3**	**A259T/D**	**A259T/D**
	A447S/V	**A543E**
	**A543E**	**D616N**
	**D616N**	D643N/E
	D643E/N	D76N/E
	D76N/E/H	**E379K**
	**E379K**	**E399K**
	**E399K**	E75K
	E75K/D	**G431E**
	**G431E**	**G525E**
	**G525E**	H38Q
	**I187K**	**I187K**
	**I218N**	**I218N**
	**I325K**	**I325K**
	I474K	**I528K**
	**I528K**	**I529K**
	**I529K**	**I644K**
	**I644K**	L143I/Stop
	**L236Q/I**	**L236Q/I**
	**L436Stop**	L378Stop
	**N186K**	**L436Stop**
	N195K	N144K
	**N432K**	**N186K**
	N51K	**N432K**
	N77K	**Q544K**
	**Q544K**	**Q548K**
	**Q548K**	**Q686K**
	**Q686K**	**R196K**
	**R196K**	**R203K**
	**R203K**	**R238K**
	**R238K**	R316K
	**R324K**	**R324K**
	**R571K**	**R571K**
	R705K	**R722K**

			**R722K**	**S359N/R**
			**S359N/R**	**Stop753K**
			**Stop753K**	**T145K**
			**T145K**	T146K
			T20K	**T361K**
			**T361K**	**T39K**
			**T39K**	**T752K**
			**T752K**	**V184I/E**
			**V184I/E**	**V252L**
			**V252L**	V450F
			**Y381N/Stop**	**Y381N/Stop**
			**Y619Stop**	**Y619Stop**
			A306T/D	E137K
			C45S	**E194K**
			C603Stop	**E224K**
			D109G	E26A
			D261N	E329G
			D670N/E	**E621K**
			E193K	**E65K**
			**E194K**	**E70K**
			**E224K**	G622E
			E23K	**I375K**
			E240K	I38T
			E260K	**M376K/I**
			E26K	N745K
			E29Stop	Q583K
			E308K	Q595K
			E311K	**Q71K**
			E333K	**R671K**
			E378K	Stop727K
			E468K	Stop742K
			E550K	**T20K**
			**E621K**	V625E
			**E65K**	W133Stop
			**E70K**	Y476N/Stop
			F36I	
			G225E	
			G498C	
			G622R/E	
			G68E	
			I239K	
		**1**	**I375K**	
			I48K	
			L249Stop	
			L35I	
			L466Stop	
			L641F	
			L668I/Stop	
			**M376K/I**	

		N332K	
		N395K	
		N467K	
		P322Q	
		P534T/Q	
		Q566K	
		**Q71K**	
		R241K	
		R634I	
		**R671K**	
		S299Y	
		S415N/R	
		S436N/R	
		**T20K**	
		T414K	
		V403D	
		V47E	
		W133R/Stop	
		W259C	
		Y476Stop	
		Y497F	
		Y683F	
		D670N/E	**E137K**
		**E137K**	**E165K**
		**E165K**	**E193K**
		**E193K**	**E194K**
		**E194K**	**E26K**
		**E26K**	**E308K**
		**E308K**	E329G
		**E333K**	**E333K**
		**E378K**	**E378K**
		**E65K**	E621K
		**E70K**	**E65K**
		E710K	**E70K**
		E726K	G225E
		F709L	G622E
**PA**		G622R/E	**I375K**
		G681E	I38T
		**I375K**	**I48K**
		**I48K**	**M376K/I**
		L249Stop	N745K
		L624I/Q	**P322Q**
		L641F	**Q583K**
		L668I/Stop	**Q595K**
	**2**	**M376K/I**	**Q71K**
		N19K	**R241K**
		N332K	**R671K**
		N395K	**S415N/R**
		N444Y	**Stop727K**

	P254H	Stop742K
	**P322Q**	**T20K**
	P534T/Q	**T414K**
	**Q583K**	**V47E**
	**Q595K**	V625E
	**Q71K**	**W133Stop**
	**R241K**	**Y476N/Stop**
	R438K	
	R439K	
	**R671K**	
	**S415N/R**	
	**Stop727K**	
	**T20K**	
	**T414K**	
	V443F	
	**V47E**	
	V625I/E	
	**W133Stop**	
	**Y476N/Stop**	
	D231E	D670E/N
	D670N/E	**E137K**
	**E137K**	**E165K**
	**E165K**	E176K
	**E193K**	**E193K**
	**E194K**	**E194K**
	**E308K**	E26K
	E378K	**E308K**
	**E65K**	E329G
	**E70K**	E468K
	**E710K**	**E65K**
	**F709L**	**E70K**
	G622R/E	**E710K**
	**G681E**	**F709L**
	I325K	G622E/R
	I347K	**G681E**
	I375K	I38T
	I48K	**L624I/Q**
	I723K	**L641F**
	**L624I/Q**	**L668I/Stop**
**3**	**L641F**	M376I/K
	**L668I/Stop**	N444Y
	M376K/I	N467K
	N19K	**P322Q**
	N395K	**P534Q**
	**P322Q**	**Q595K**
	**P534Q**	**Q71K**
	Q583K	**R241K**
	**Q595K**	**R438K**
	**Q71K**	**R439K**

		**R241K**	**R671K**
		**R438K**	**S415N/R**
		**R439K**	**Stop727K**
		**R671K**	**T20K**
		**S415N/R**	**T414K**
		**Stop727K**	V443F
		**T20K**	V625E/I
		**T414K**	**W133Stop**
		V625I/E	**Y476N/Stop**
		**W133Stop**	
		**Y476N/Stop**	
**HA1**	**1**	A154E	**E183K**
		D100E	**E184K**
		D185E	N165K
		**E183K**	**N303K**
		**E184K**	**T47K**
		E86K	
		G218V	
		G258E/R	
		K344Stop	
		L343Stop	
		**N303K**	
		P171Q	
		P269R	
		P31Q	
		Q186K	
		R101K	
		S148N/R	
		T102K	
		T147N	
		T221S	
		T257K	
		T259K	
		**T47K**	
	**2**	**D100E**	**D100E**
		**D163E**	**D163E**
		**E183K**	D185E/N
		**E184K**	**E183K**
		**E232K**	**E184K**
		**E235K**	**E232K**
		**N164K**	**E235K**
		**N165K**	**N164K**
		**N303K**	**N165K**
		P36Q	**N303K**
		**Q233K**	Q186K
		**Q263K**	**Q233K**
		**R101K**	**Q263K**
		**T102K**	**R101K**
		T234K	S148R

		**T37K**	**T102K**
		**T47K**	**T37K**
			**T47K**
	3	**D100E**	A42E
		**D163E**	**D100E**
		**E183K**	**D163E**
		**E184K**	D185E/N
		**E232K**	**E183K**
		**E235K**	**E184K**
		I7K	**E232K**
		**N164K**	**E235K**
		**N165K**	**N164K**
		**Q186K**	**N165K**
		**Q233K**	P36T/Q
		Q263K	**Q186K**
		**R101K**	**Q233K**
		**T102K**	**R101K**
		**T37K**	**T102K**
		**T47K**	T234K
			**T37K**
			**T47K**
	1	C219F	H142Q
		E105K	**I107K**
		E43K	**I108K**
		E82K	**I48K**
		E97K	**T49K**
		G106E/R	**V217F**
		G225E	
		**I107K**	
		**I108K**	
		I208F	
		**I48K**	
		I96K	
		M211L	
		N46K	
		Q42K	
		Q95K	
		S163F	
		S196Y	
		S220Y	
		**T49K**	
		V131I	
		V200E	
		V209F	
		**V217F**	
		V60E/I	
		Y210F	
		**D215E/N**	**D215E/N**
		**E43K**	**E43K**

HA2	2	**E97K**	**E97K**
		**G106E/R**	**G106E/R**
		G225E	**H142N/Q**
		**H142N/Q**	**I107K**
		**I107K**	**I108K**
		**I108K**	I208F
		**I48K**	**I48K**
		**I96K**	**I96K**
		M211L	**N46K**
		**N46K**	**Q42K**
		**Q42K**	**Q95K**
		**Q95K**	**T49K**
		**T49K**	**V200E**
		V131I	**V217F**
		**V200E**	V60E/I
		**V217F**	
		Y210F	
	3	**D215E/N**	**D215E/N**
		**E43K**	**E43K**
		**E97K**	**E97K**
		**G106E/R**	**G106E/R**
		**H142N/Q**	G225E
		**I107K**	**H142N/Q**
		**I108K**	**I107K**
		**I48K**	**I108K**
		**I96K**	I208F
		**N46K**	**I48K**
		**Q42K**	**I96K**
		**Q95K**	M211L
		**T49K**	**N46K**
		V131I	**Q42K**
		V200E	**Q95K**
		**V209F**	**T49K**
		**V217F**	**V209F**
		V60E/I	**V217F**
			Y210F
		A366E	D161E
		D566H	**E140K**
		E104K	E278K
		**E140K**	E88K
		E74K	**I75K**
		E88D	N43K
		G193E	T244N
		G219E	L592V
		G246E	
		G259E	
		I271N	
		I565K	
		**I75K**	

		L194Q	
		L558H	
		M94L	
		N499K	
		N563K	
		P326H	
	**1**	P72T/Q	
		Q92K	
		R105K	
		R236Stop	
		R325M	
		R373K	
		R482K	
		R483K	
		S212Stop	
		S330N/R	
		S42R	
		S562T	
		Stop561K	
		T122S	
		T73K	
		V138F	
		V328E	
		V355D	
		V481I/E	
		Y560N/Stop	
		**D161N/E**	S42A
		**D380E**	**N43K**
		**E278K**	**E74K**
		**E74K**	**I75K**
		**E88K**	**E88K**
		**G219E**	H147Q
		**I75K**	**N148K**
**NP**		**L194Q**	**D161N/E**
		**L372I/Stop**	R170K
		L414I/Stop	E176K
		**N148K**	T180K
		**N286K**	**L194Q**
		**N43K**	**G219E**
	**2**	R105K	**E278K**
		R304K	**N286K**
		**R373K**	**S303R**
		**R482K**	**L372I/Stop**
		**R483K**	**R373K**
		**S303R**	**D380E**
		S42R	**R482K**
		Y560N	**R483K**
			V496D
			Y560N/Stop

				Stop561K
				S562R
				N563K
			D15E	S42A
			**D161N/E**	**N43K**
			**D380E**	**E74K**
			**E143K**	**I75K**
			**E144K**	**E88K**
			**E278K**	R105K
			**E74K**	**E143K**
			**E88K**	**E144K**
			**G219E**	H147Q
			I169K	**N148K**
			**I75K**	**D161N/E**
			**L194Q**	**R170K**
			L403Stop	T180K
		**3**	**L414I/Stop**	**L194Q**
			**N148K**	**G219E**
			**N43K**	R235K
			P72Q	**E278K**
			R116K	S303R
			**R170K**	R304K
			R482K	L372I/Stop
			R483K	R373K
			S42R	**D380E**
			**Stop561K**	Q383K
			Y277Stop	**L414I/Stop**
			**Y560N/Stop**	**Y560N/Stop**
				**Stop561K**
				N563K
			Y30N	**T43K**
			L38Q	**E44K**
			S41Stop	**I45K**
			S42T	**G108R/E**
			**T43K**	**R147K**
			**E44K**	**E148K**
			**I45K**	D149N/E
			E105K	**R150K**
			**G108R/E**	**N151K**
			I115K	**I204K**
			P124Q	N219K
			G145V	**E226K**
			**R147K**	**I262K**
			**E148K**	**I262K**
			**R150K**	**N284K**
			**N151K**	**I333K**
			K152N	
			P165Q	
			**I204K**	

NA	1	T213R	
		Y214N	
		L222Q	
		T224K	
		Q225K	
		**E226K**	
		Y237F	
		L238F	
		I261K	
		**I262K**	
		P267S	
		E272K	
		E275K	
		E276K	
		**N284K**	
		A298T/E	
		L305Stop	
		S332R	
		**I333K**	
		Q355K	
		R356K	
		R367Q	
		T368K	
		I372N	
	2	Stop39K	L38Q
		H42Q	S42T/Stop
		N44Q	T43K
		Stop46K	E44K
		Y86F	I45K
		P107T/Q	L85F
		**G108R/E**	T106N
		**I114K**	**G108R/E**
		**I115K**	**I114K**
		D194N/E	**I115K**
		**R150K**	D149N
		**N151K**	**R150K**
		**I204K**	**N151K**
		**N219K**	**I204K**
		**Y237F**	**N219K**
		**L238F**	**Y237F**
		**I261K**	**L238F**
		**I262K**	**I261K**
		**E264K**	**I262K**
		**N284K**	**E264K**
		**A298T/E**	**N284K**
		L305I/Stop	**A298T/E**
		**I333K**	L305T/Stop
		**R356K**	**I333K**
		L372Q	**R356K**

		**I415K**	**I415K**
		**D417N/E**	**D417N/E**
		E418K	
	**3**	**L38Q**	**L38Q**
		**S42T/Stop**	**S42T/Stop**
		**T43K**	**T43K**
		**E44K**	**E44K**
		**I45K**	**I45K**
		**L85F**	**L85F**
		**G108R/E**	**G108R/E**
		**I114K**	**I114K**
		**I115K**	**I115K**
		**E148K**	**E148K**
		**D149E**	**D149E**
		**R150K**	**R150K**
		**N151K**	**N151K**
		**I204K**	**I204K**
		**E226K**	**E226K**
		**Y237F**	**Y237F**
		**I261K**	**I261K**
		**I262K**	**I262K**
		**E264K**	**E264K**
		**N284K**	**N284K**
		**L305I/Stop**	**L305I/Stop**
		**I333K**	**I333K**
**NB**	**1**	I32K	**N45K**
		F43L	**R46K**
		I44N	**N47K**
		**N45K**	
		**R46K**	
		**N47K**	
	**2**	**I44N**	**I44N**
		**N45K**	**N45K**
		**R46K**	**R46K**
		**N47K**	**N47K**
		**I88F**	**I88F**
	**3**	**I44N**	**I44N**
		**N45K**	**N45K**
		**R46K**	**R46K**
		**N47K**	**N47K**
		**I88F**	**I88F**
		E196K	E23K
		E36K	**G191E**
		**G191E**	**Q154K**
		G193V	**T91K**
		I46K	
		I55K	
		K68I/Stop	
		L67F/Stop	

	**1**	M177L	
		N48K	
		**Q154K**	
		Q219K	
		Q56K	
		R74K	
		S218R	
		T88K	
		**T91K**	
	**2**	**A22E**	**A22E**
		**A25E/T**	**A25E/T**
		**E23K**	D71Y
		**E26K**	**E23K**
		**I46K**	**E26K**
		**L24I/Q**	G191E
		**N48K**	**I46K**
		**Q154K**	K68I/Stop
**M**		**Q219K**	**L24I/Q**
		R74K	L67F
		**S218R**	**N48K**
		**T88K**	**Q154K**
		**T91K**	**Q219K**
			**S218R**
			**T88K**
			**T91K**
	**3**	**A22E**	**A22E**
		**A25E/T**	**A25E/T**
		**E23K**	A90E
		**E26K**	D71Y
		**G191E**	**E23K**
		**I46K**	**E26K**
		**K68I/Stop**	**G191E**
		L24Q	**I46K**
		**L67F**	**K68I/Stop**
		**N48K**	L24I/Q
		**Q154K**	**L67F**
		**Q219K**	**N48K**
		**R101K**	**Q154K**
		R74K	**Q219K**
		**S218R**	**R101K**
		**T88K**	**S218R**
		**T91K**	**T88K**
			**T91K**
		E104Stop	D47E
		E63K	E106K
		E79D	E107K
		E93D	L15Stop
		H19N	**T48K**
		H84Q	

		I14F	
		I49K	
	1	I95K	
		I96K	
		L103F	
		M37I	
		Q61K	
		S91F	
		**T48K**	
		T69K	
		Y60Stop	
		E63K	C11F
**MB**		**I49K**	D47E
		L10F	E106K
		**M37I**	E107K
		**Q61K**	**I49K**
	2	R106K	L114Stop
		R107K	L15Stop
		**T48K**	**M37I**
		**Y60Stop**	**Q61K**
			**T48K**
			**Y60Stop**
		**D47E**	**D47E**
		**E63K**	E106K
		L15Stop	E107K
		**M37I**	**E63K**
	3	N48K	I49K
		**Q61K**	L110Stop
		R106K	**M37I**
		R107K	**Q61K**
		**T48K**	**T48K**
		**Y60Stop**	**Y60Stop**
		A257E/G	L51Q
		A258S	N151K
		A95S	**N152K**
		E144K	R53K
		E168K	R58K
		E245D	**V163I/E**
		E249D	E259K
		E32K	
		F100V	
		I159K	
		I175K	
		I255L	
		K253N	
		L185F	
	1	**N152K**	
		P142Q	
		P254Q	

		Q157K	
		R217T	
		S252Stop	
		T174K	
		T260S	
		**V163I/E**	
		V262G	
		V82I/E	
		W36R/C	
**NS1**		Y116Stop	
		Y121C	
		Y31Stop	
		E168K	E259K
		**I159K**	**I159K**
		**I175K**	**I175K**
		**I59K**	**I59K**
		**L51I/Q**	**L51I/Q**
	**2**	**N151K**	**N151K**
		**N152K**	**N152K**
		**R53K**	**R53K**
		**R58K**	**R58K**
		**S57Stop**	**S57Stop**
		S69N/R	S69R
		E168K	E259K
		**I159K**	**I159K**
		**I175K**	**I175K**
		**I59K**	**I59K**
		**L51I/Q**	**L51I/Q**
		**N151K**	**N151K**
		**N152K**	**N152K**
	**3**	Q214K	**R53K**
		**R53K**	**R58K**
		**R58K**	**S57Stop**
		**S57Stop**	**S69N/R**
		**S69N/R**	V163I
		V163I/E	
		D78Y	D79E
		D79E/N	**I93K**
		D96Y	
		F37Y	
		**I93K**	
		K31T	
		K41N	
		L29Stop	
	**1**	L82F	
		Q34L/K	
		Q36H/K	
**NS1**		Q39K/E/H	
		R76K	

		S27I	
		S35I	
		V87E	
		W44G	
	**2**	**D79E**	**D79E**
		**E259K**	**I93K**
		**I93K**	**L72F**
		**L73F**	**L73F**
		**Y71F**	**Y71F**
	**3**	**D79E**	**D79E**
			I93K

*Bolded substitutions indicate common substitutions between WT and mutant virus

†N/A: Not applicable; No substitutions were observed in the viral protein after passage
